# Revolutionizing drug development: harnessing the potential of organ-on-chip technology for disease modeling and drug discovery

**DOI:** 10.3389/fphar.2023.1139229

**Published:** 2023-04-25

**Authors:** Naina Sunildutt, Pratibha Parihar, Abdul Rahim Chethikkattuveli Salih, Sang Ho Lee, Kyung Hyun Choi

**Affiliations:** ^1^ Department of Mechatronics Engineering, Jeju National University, Jeju, Republic of Korea; ^2^ College of Pharmacy, Jeju National University, Jeju, Republic of Korea

**Keywords:** organ on a chip (OCC), disease modelling, parameters, drug discovery, pharmacokinetic & pharmacodynamic parameters

## Abstract

The inefficiency of existing animal models to precisely predict human pharmacological effects is the root reason for drug development failure. Microphysiological system/organ-on-a-chip technology (organ-on-a-chip platform) is a microfluidic device cultured with human living cells under specific organ shear stress which can faithfully replicate human organ-body level pathophysiology. This emerging organ-on-chip platform can be a remarkable alternative for animal models with a broad range of purposes in drug testing and precision medicine. Here, we review the parameters employed in using organ on chip platform as a plot mimic diseases, genetic disorders, drug toxicity effects in different organs, biomarker identification, and drug discoveries. Additionally, we address the current challenges of the organ-on-chip platform that should be overcome to be accepted by drug regulatory agencies and pharmaceutical industries. Moreover, we highlight the future direction of the organ-on-chip platform parameters for enhancing and accelerating drug discoveries and personalized medicine.

## Introduction

The pharmaceutical industries and regulatory agencies are constantly considering a highly efficient platform for alternative animal studies. Recently, the US Senate unanimously passed the Food and Drug Administration (FDA) Modernization Act (Han, 2023), a bill that grants permission to evaluate experimental drugs using cutting-edge non-animal alternatives for more scientifically significant platforms. Animal models have traditionally been employed in disease modeling and drug discovery, to find novel therapeutic targets, toxicity evaluation, efficacy, and drug dosing. However, the major challenges for drug development are the lack of human pathophysiologically relevant outcomes and reproducibility in the current *in vitro* two-dimensional and animal models. These limitations can be overcome using promising organ-on-chip platforms, which is a novel technology that has the potential to replace *in vivo* animal models with complex advanced *in vitro* models. This complex microphysiological system has evolved as a powerful next-generation *in vitro* culture platform to accurately mimic the human body, providing insights into the different organ pathophysiology and functionality, thereby acting as an ultimate platform for drug testing and development.

The features of the organ on chip platforms are small microchannels with living human tissues and cells under controlled dynamic microfluidic culture. Moreover, it can also control critical parameters such as shear stress, cell-cell/tissue-tissue interactions, cell patterning, concentration gradients, mechanical cues, and tissue boundaries, which are essential to accurately mimic human organ models, physiology, and disease conditions. A multi-organ chip system is created when more than two organ chips are fluidically connected. By expanding this system, a human body-on-chips can be created, simulating the entire body’s pathophysiology and drug absorption, distribution, metabolism, and excretion (ADME). The combination of organ-on-chip platform and patient-specific induced pluripotent stem cells (iPSCs) can develop better human organ pathophysiological environments and ideal disease models. iPSC differentiation has the potential to enhance both the personalized medicine and the translational value of organ on chip platform.

Prior research has focused on designing and optimizing the organ on chip platforms and experimental evidence of organ on chip platform’s capacity to mimic organ functions and the human pathophysiological environment Despite the limitations of existing organ-on-chip platforms, efforts have been focused on replicating key physiological features of specific organs in order to effectively replace the use of animal models. In order to surpass animal models, scientists redirected their attention towards emulating the vital elements of a particular organ on a microfluidic chip. If scientists can accurately simulate the pathophysiology of human organs using organ-on-chip technology, it would facilitate the investigation of disease-specific pathways and contribute to the advancement of personalized medicine. The FDA will approve it for use in clinical trials and subsequent disease investigations, allowing them to expedite the process and save many steps in between. Following this, pharmaceutical companies can accelerate the drug discovery and development process.

Here in this review, we first discuss the different disease phenotypes such as genetic diseases, cancers, infectious diseases, incurable diseases, etc., and their parameters that have been previously modeled in organ on chip platform. In the following section of the review, we introduce the recent and past drug discoveries as well as toxicity testing in organ on chip platform. Additionally, we mention the biomarkers and assays used for the confirmation of various diseases in the organ on chip platform.

### Conventional disease models and their challenges

Animal or cell models are frequently used by researchers to study the progression of human diseases and test new potential therapies. Animal models are crucial for understanding disease pathophysiology and evaluating novel treatments. However, they fall short of predicting the effectiveness and safety of many pharmaceuticals in clinical trials (McGonigle and Ruggeri, 2014). Additionally, the pursuit of alternatives has been triggered by the ethical concerns associated with animal testing. These problems could be resolved using cell models. However, they are unable to replicate the intricate connections between various cell types in tissues and organs within the human body (Low et al., 2021).

### Disease modeling in organ on chip platform

Organ on chip platforms are bioengineered microdevices that imitate the fundamental functions of organs and tissues. They consist of a variety of cell types to accurately represent the physiological balance and the crucial biomechanical forces acting on the mimicked tissues (Low et al., 2021). Organ on-chip platforms have several advantages over conventional disease models, the most significant of which is their capacity to manipulate the cellular and tissue milieu, bio-mechanical and biochemical forces to mimic in human responses. Additionally, by vascularizing or perfusing tissues, researchers can provide cultured cells with nutrients and fluid flow. Ultimately, they can integrate real-time sensors to track the condition and activity of the cells (Low et al., 2021).

### Organ on chip platform holistic strategy for disease modeling

The human organs are physically separated, but they can still communicate with one another through blood and lymphatic circulation to maintain the body’s equilibrium. Multiple organ interactions are essential for the proper functioning of the body. The small intestine, for instance, absorbs the things that have been digested, the liver breaks them down, the bloodstream carries them to the appropriate organs, and the kidneys eliminate waste. Drug reactions in our body are influenced by this intricate cycle of absorption, distribution, metabolism, and excretion. Additionally, numerous physiological activities within the endocrine system depend on regulatory pathways and hormonal feedback loops. Therefore, systemic organ communication is essential to describe and reproduce human physiological functioning. A more thorough systemic approach is also required because many diseases, such as cancer, osteoarthritis, and metabolic diseases, affect many organs. Thus, researchers created organ on chip platform to simulate multiple organs in a single device (Picollet-D’hahan et al., 2021).

### Applications for multiple organ on chip platform

Using Multi-organ on chip techniques, researchers can discover the crucial molecular mechanisms underlying complex diseases (Picollet-D’hahan et al., 2021). A multi-organ on chip system that simulates several aspects of the brain is one example. Employing this technology, researchers were able to comprehend how blood-brain barrier (BBB) microvascular cells and neurons communicate metabolically (Maoz et al., 2018). Using the same method, a different study demonstrated type 2 diabetes. Although human pancreas and liver cells successfully retained blood postprandial glucose levels when grown separately, the glucose levels in both organ modules remained high (Bauer et al., 2017). Among other potential uses, multi-organ on chip platform can be utilized to investigate cancer metastasis and the reproductive function of women (Low et al., 2021).

### Fundamental principles of organ on chip platform

Recent developments in micro-nanotechnology (especially microfluidics), physiology, cellular biology, and tissue engineering have led to the development of the technology for organ on chip platform (Li et al., 2019). It is driven by the requirement for affordable, trustworthy *in vitro* models that could replace animals during the most labor-intensive and costly phases of the development of products (Busek et al., 2022). For the enhanced understanding of the pathophysiological processes involving the interplay of tissue and organs, exogenous stimuli, and immune system (such as nutraceuticals and pharmaceuticals) both in disease and healthy states, organ on chip platform technology intends to create efficient and transferrable interconnected microphysiological models. This can be attained by replicating the basic function and architecture of a single human cell-tissue or a functional organ *in vitro*. Basic cellular biology and a wide range of implementations, such as networks of neuron, drug screening, engineering of tissues, and cell-based biosensors, frequently make use of cell patterning on microfluidic chips (Wu et al., 2018; Zhao et al., 2020). The significance of fluid dynamics in organ on chip technology cannot be overstated, as it plays a critical role in disease modeling and drug testing. Reynolds number is a crucial parameter in microfluidics, as it determines whether the flow is laminar or turbulent. Laminar flow is desirable in organ-on-chip technology as it provides a consistent flow rate and avoids disturbances in the microenvironment (Regmi et al., 2022; Lingadahalli Kotreshappa et al., 2023). Scaling concepts such as the hydraulic diameter, flow rate, and shear stress are essential for designing and optimizing organ-on-chip devices (Wikswo et al., 2013). Other important parameters in microfluidics include pressure, flow rate, viscosity, surface tension, and wettability. These parameters can have a significant impact on the behavior of fluids in microfluidic devices, and thus on disease modeling and drug testing (Preetam et al., 2022). For example, variations in flow rate and pressure can affect the shear stress experienced by cells in microfluidic channels, which can in turn influence their behavior and function. Similarly, surface tension and wettability can impact the adhesion and migration of cells within microfluidic devices, which is important for studying processes such as angiogenesis and tumor metastasis (Tovar-Lopez et al., 2019; Kausar et al., 2021; Farooqi et al., 2022; Kausar et al., 2022). These parameters enable the selection of appropriate materials, cell types, and culture conditions to ensure optimal organ functionality and accurate disease modeling (Ahmed et al., 2022a; Ahmed, 2022).

**FIGURE 1:**
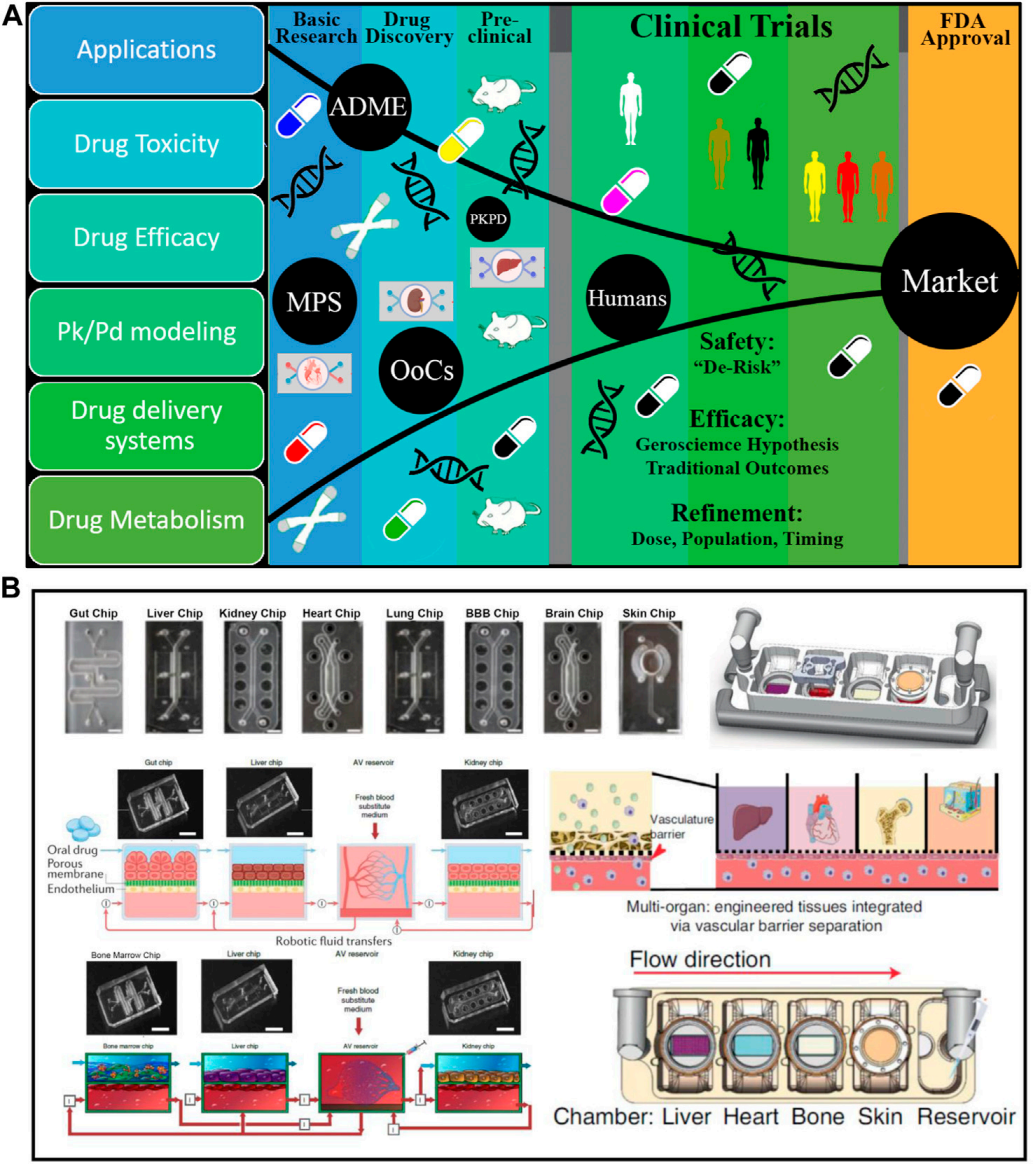
Microphysiological System-Organ on a Chip (organ on chip platform) Applications, Drug Discovery, and platforms. **(A)** Application of organ on chip platform in drug discovery includes the drug toxicity, efficacy, modeling of PK-PD, drug delivery, and drug metabolism studies. organ on chip platform helps to accelerate every stage of the drug discovery process from the basic research to clinical trials with the least failure of drugs compare to current animal models. Additionally, no ethical issues, cost effective, short time, and human relevant outcomes. **(B)** organ on chip platform designs for single organs and multi-Organ platforms: Vascularized multiorgan-on-organ on chip platform by integrating gut chip, liver chip, kidney chip, heart chip, lung chip, BBB chip, brain, chip and skin chip. Vascular barrier is integrated through vascular barrier separation as observed *in vivo*. A vascular barrier underneath each tissue allows integration, creating a tissue-specific niche in the upper chambers for each created organ while facilitating crosstalk across organs within the system via vascular perfusion. Diagram of interconnected two-channel organ on chip platform with endothelium-lined circulation channels and parenchymal cells that are fluidically linked with one another and an arteriovenous mixing reservoir utilizing a robotic liquid handler. The reservoir is built into a system to simulate the mixing of blood in the central circulation and to enable fluid sampling that is more similar to taking peripheral blood from a patient. ADME, absorption, distribution, metabolism and excretion; PKPD, pharmacokinetic/pharmacodynamic; MPS, Microphysiological Systems; OoCs, organ-on-a-chip; FDA, Food and Drug Administration (Novak et al., 2019; Herland et al., 2020; Ingber, 2022; Ronaldson-Bouchard et al., 2022).

The idea of abiding co-culturing of cells in perfusion in organ on chip platform, mimicking organ on chip platform, was first put forth by the Schuler and Ingber groups with the intention of accelerating the drug development process and ultimately substituting animal testing for personalized medicine and drug development with a more precise and economically in 3D complex *in vitro* platform (Polini and Moroni, 2021). Establishing physiologically accurate 3D complex *in vitro* disease models that accurately replicate the complex pathophysiological model of the human body would be made possible by organ on chip platform technologies. Although the status of microfluidic technology as it is used in research labs today falls short of this goal (Maschmeyer and Kakava, 2022). The technological disparity between animal models and clinically-industrially acceptable models still exists despite the fact that many research groups around the world have made considerable progress to create *in vitro* organ on chip platform models of numerous organs or tissues, including the kidney, gut, lung, bone, brain/BBB (blood-brain barrier), liver, vasculature, heart, and diseases like thrombosis, tumor, and infection (Picollet-D’hahan et al., 2021). organ on chip platform systems are anticipated to be created utilizing more than 90% of the microfluidic chips at present on the market, which consist of a porous membrane and one or more single or double-layered channels connecting them. This type of gadget was created in academic research labs or by small enterprises that lacked the funding necessary to invest in the engineering systems needed to create organ on chip platform (Sontheimer-Phelps et al., 2019; van Berlo et al., 2021). Even though there have been a lot of studies on organ on chip platform published over the past 10 years, only rare biological data are now accessible to address this problem. These results primarily highlight the use of more than one type of cells cocultured, as a miniature for a particular human organ-tissue, simple functional assays and specific characterization of tissue-tissue/cell-cell communications (Ahmed, 2022). These research assessed the current breakthroughs in organ on chip platform, recognized new limitations that must be conquered to reorganize organ on chip platform models into respectable human Pathophysiologically relevant models, and discussed potential avenues of research to pursue in this area in the future (Costa et al., 2020; Ramadan and Zourob, 2020). According to Skardal *et al.* remarkable improvement has been accomplished in the design and use of complex multi-organ on chip platform and body on a chip, in addition to the implementation of this organ on chip platform technology for drug efficacy and toxicity testing, personalized medicine and disease modeling (Figure 1A) (Skardal et al., 2016). While discussing the possible uses of organ on chip platform technology in individualized and precision medicine, van den Berg *et al.* also highlighted the use of machine learning techniques and timelapse microscopy for the development of organ on chip platform technology (Bhatia and Ingber, 2014; Mencattini et al., 2019; Ahmed et al., 2022b). A thorough assessment of the advancement in organ on chip platform research finds that “X-on-a-chip” (where “X" refers for a tissue or organ) proof-of-principle studies focused on duplicating a particular organ or tissue-like structure *in vitro* continue to dominate this technology’s state of art (Polini and Moroni, 2021). A common chip design and methodology are used in the majority of these investigations, which entail co-culturing relevant cells near to one another and altering the cell types for each (Martinez et al., 2019; Schuster et al., 2020). These tests were all conducted using the same chip and techniques. With the exception of a few prominent outliers, dedicated and significant research on a single organ is still woefully underrepresented. It is crucial to fully understand the cell-cell communication as well as the basic tissue functions and architecture of a specific organ before incorporating it with a different working organ (Hoogduijn et al., 2020; Schuster et al., 2020). Cell biology, biomarker identification and device engineering must be combined in order to generate a fully functional organ on chip platform in 3D complex *in vitro* model (Holloway et al., 2021; Polini and Moroni, 2021). The creation of reliable *in vitro* models would be endangered by a lack of advancement in these areas, which would be harmful to the field. The development of the organ on chip platform technology will be covered in the section that follows (Ahmed, 2022).

### Microengineering of organ on chip platform

As seen by the expansion of at least 28 organ on chip platform companies in less than 7 years, organ on chip platform engineering has attracted significant interest from pharmaceutical corporations, regulatory bodies, and even national security agencies (Balijepalli and Sivaramakrishan, 2017). organ on chip platform technology is primarily focused on three areas: the organ multicellular interfaces (such as gut, lung and blood vessel networks), the arrangement of parenchymal cells at the tissue level (such as the such as the heart and liver), and the organs’ diastolic and systolic systems, which act as tissue barriers in organs like the liver and kidneys (Huh et al., 2013; Peck et al., 2020; Garcia-Gutierrez and Cotter, 2022). The challenges that have to be overcome to bring the chips from an idea to reality are highlighted in the following sections.

## Challenges faced in organ on chip platform development

### Organ on chip platform development challenges in terms of engineering

Organs on a chip are created by combining microfabrication technologies developed for the semiconductor industry with cell and micro tissue culture (Garcia-Gutierrez and Cotter, 2022). The latter is a three-dimensional, organic universe, whereas the former is a flat, unchanging universe (Carvalho et al., 2021). Problems arise when cells are forced to co-exist and develop with non-biological components or methods. As engineers develop organ on chip platform, several aspects of the development phases and designs should be taken into account (Mastrangeli et al., 2019; Semertzidou et al., 2020; Fritschen and Blaeser, 2021; Lam et al., 2021).

### Designing problems for MPS-OCC

During the modelling stages, organ-miniatures are developed that can be utilized to address biological dilemmas (Sung, 2021). Micropumps and actuators could be used with structured soft materials to mimic the basic movements and pumping actions of organs (Lam et al., 2021). In a lung-on-a-chip system, a microcavity develops that exposes the lung epithelium to airflow (Meghani et al., 2020; Rokhsar Talabazar et al., 2021; Liu et al., 2022b). A kidney-on-a-chip platform has a porous and thin membrane separating two flows of urine and blood, and the kidney epithelium filters toxins through this barrier (Miccoli et al., 2018; House et al., 2021). The planar limitations of microfabrication are employed in these designs to produce simple but useful models. Scaling arguments also play a critical role in the design of microfluidic chips for disease modeling and drug testing. Other key considerations include technical limitations and the relevance of the chip to the targeted organ. In addition to similarity and relevance with the targeted organ and technical limitations, scaling arguments during chip design also consider factors such as the size and shape of the microchannels, the material properties of the chip, and the fluid flow patterns within the chip. For example, microchannels must be small enough to facilitate laminar flow and allow for precise control of fluid behavior, but not so small that they become clogged or prohibit efficient mixing. Additionally, the material properties of the chip must be carefully selected to ensure compatibility with the fluids being used, and to minimize interactions between the chip and the fluid that could lead to inaccurate results. Furthermore, scaling arguments must also take into account the overall complexity of the model being developed. For example, if a model requires multiple cell types and complex interactions between those cells, then the design of the chip must be able to accommodate this complexity. This may involve creating more intricate channel geometries, or using more advanced fabrication techniques to create multi-layered chips (Ahmed, 2022).

In addition to controlling the movement, location, and morphology of growing cells, organ on chip platform must be capable of controlling basic tissue or organ function; if not, the cell phenotype may not be maintained. These models are built with the aid of microfabrication’s planar constraints. Along with controlling basic organ or tissue function, it is also necessary to manage the movement, location, and form of growing cells. The planar limitations of microfabrication are applied to the construction of these models. Along with controlling basic organ or tissue function, growing cells’ positioning, mobility, and shape must also be regulated. The model should measure important parameters including cell activity and secretion in addition to simulating the organ’s operations. There are various monitoring options for each metric. Currently, not all techniques are suitable due to sensitivity or speed. There are advantages and disadvantages to both electrochemical and optical methods for measuring pH. For each trial, designers must select the best option. First, figure out how to imitate organ structure, function, retain cell features, and evaluate important metrics (Ahmed, 2022).

### Manufacturing challenges of organ on chip platform

On a millimeter scale, processes including vasculature, filtration, and separation take place (Santoso and McCain, 2020; Zheng et al., 2021). With the same resolution, an organ on chip platform can perform analogous functions. The only manufacturing method that can produce features with constant proportions on such tiny sizes at this time is microelectronics. Electronic parts and sensors on silicon wafers are the result of years of sub-micron etching and material deposition (Albanese et al., 2013; Stucki et al., 2018; Chen et al., 2021a; Nelson et al., 2021; Soomro et al., 2022). organ on chip platform and microfluidic chips were built using the same microfabrication. Microfabrication is done in a cleanroom with high operating costs to prevent contamination of the tools and materials (Bhatia and Ingber, 2014; Kurth et al., 2020; Sun et al., 2020). The master of glass or silicon was made in a sterile environment, then it was shaped and replicated externally utilizing economically and environment friendly materials such as Polydimethylsiloxane (PDMS). Because of their sensitivity to chemicals, medications can accidentally get trapped or released, which is a severe problem that needs to be handled. Facilities for microfabrication employ quality control practices that might or might not be appropriate for cell culture applications. As industrialization and commercialization advance, the importance of creating organs-on-a-chip may rise (Sitti and Wiersma, 2020; Convery et al., 2021; Jin et al., 2021; Nitsche et al., 2022). Engineers are looking into new fabrication processes and materials due to the limits of current fabrication techniques. Different additive methods of fabrication like 3D dispenser inkjet printing, which have been accurately positioning microdroplets for years (Miller et al., 2020; Sitti and Wiersma, 2020; Sun et al., 2020). Functional biomaterials like collagen, proteins, and even living cells can currently be printed using printers. Early-stage bioprinting businesses RegenHU (Switzerland) and Cellink (Sweden) are concentrating on tissue culture and organ on chip platform applications, making these high-tech breakthroughs further accessible to researchers and engineers. Although 3D printing can be used to create entire devices, the technology is currently too sluggish to compete with microfabrication. Microfabrication’s manufacturing scale and 3D printing’s resources and flexibility could be coupled to yield an interesting and practical solution (Achberger et al., 2019; Mosavati et al., 2020; Kim et al., 2021a; Gough et al., 2021; Ahmed et al., 2022c). When necessary, 3D printers could integrate bio-functionalization to mechanical constructions mass-produced in clean rooms with electrical functionality. In contrast to the inadequate infrastructure-intensive and constrained manufacturing processes previously accessible, engineers can currently create an organ on chip platform applying more versatile and adaptable techniques. To develop an organ on a chip, surface chemistry and materials could be employed (Lai et al., 2021; Wiedenmann et al., 2021).

## Design and materials

The design process for an organ on chip platform can be difficult because these devices are made up of several components that are specifically suited to the diverse functions envisioned. When constructing microfluidic architecture, factors like size, channel number, geometry, 2-Dimentional/3-Dimentional structure, and pattern should be taken into consideration (Zhou et al., 2020a; Zhou et al., 2020b; Mosavati et al., 2020; Wagner and Radisic, 2021). These elements may have a sizable effect on the biochemical milieu that underlies cell and tissue function. Bio-surfaces such as membranes are often used to better simulate the modeled organ/tissue (Zhou et al., 2020a; Sasserath et al., 2020; Ferreira et al., 2021; Silvani et al., 2021; Stengelin et al., 2022). Additionally, they are employed for monitoring significant physicochemical variables and simulating *in-vivo* conditions. Finally, the ease of implementation and the chip’s operating conditions should be taken into account when selecting the chip-to-world interfaces, such as electrical interconnection and fluid flow (Peck et al., 2020; Görgens et al., 2021; Liu et al., 2022a). Channels are present in a microfluidic device. In a mini bioreactor, the channel network enables scaffold construction and spatial control of the development of tissues or organs. Biological tissue determines whether cells are cultured in a monolayer, multilayer, or three-dimensional bioreactor. Because of its microchannels and control of fluid, the cell culture reactor can generate the standard biochemical milieu for the enhancement and study of organs and tissues. Cells are either cultured in a mono-layered, multi-layered, or three-dimensional bioreactor depending on the biological tissue. By combining bio interfaces and pathways and ensuring that the modifications created a multilayer integrated hybrid system, it is possible to produce microarchitecture that resembles that found *in vivo*. Another aspect of microfluidic architecture and design is channel patterning (e.g., microgrooves). Geometric channel functionalization provides an environment for interface’s bio-functionalization (Ahmed, 2022). The planned simulated environment and fluid movement both affect the channel morphology. Plastic membranes are frequently used in microfluidic organ on chip platform devices to represent different barriers as well as tissue communications. In some cases, a membrane with pores is utilized to more closely resemble the characteristics of an organ or tissue. Alternative method is the placing of a porous membrane that permits the expansion of two different cell types on both sides. Two-layer channel design exposes every single type of cell to a unique milieu. Models of this architecture can be found in the liver, skin, brain, heart, lungs, and stomach (Figure 1B). organ on chip platform design is directly associated to the material utilized. The fluids, more cells, and extras such as protein fibers and minerals surround and surround the cells. A robust framework is needed to culture cells and investigate the outcomes of living model in an *in-vitro* environment (Guo et al., 2018; Lin et al., 2020; Hansen et al., 2022). In preparation, chips are composed of a mass quantity of materials to strengthen the structure and other materials for bio surface characteristics and functional alteration. Only a limited number of materials may be employed to make an organ on chip platform device due to the system complexity (Ragelle et al., 2020; Boeri et al., 2021; Guttenplan et al., 2021; Lu and Radisic, 2021; Ratri et al., 2021; Kavand et al., 2022).

### Evolving human 3D complex *in vitro* models

**FIGURE 2:**
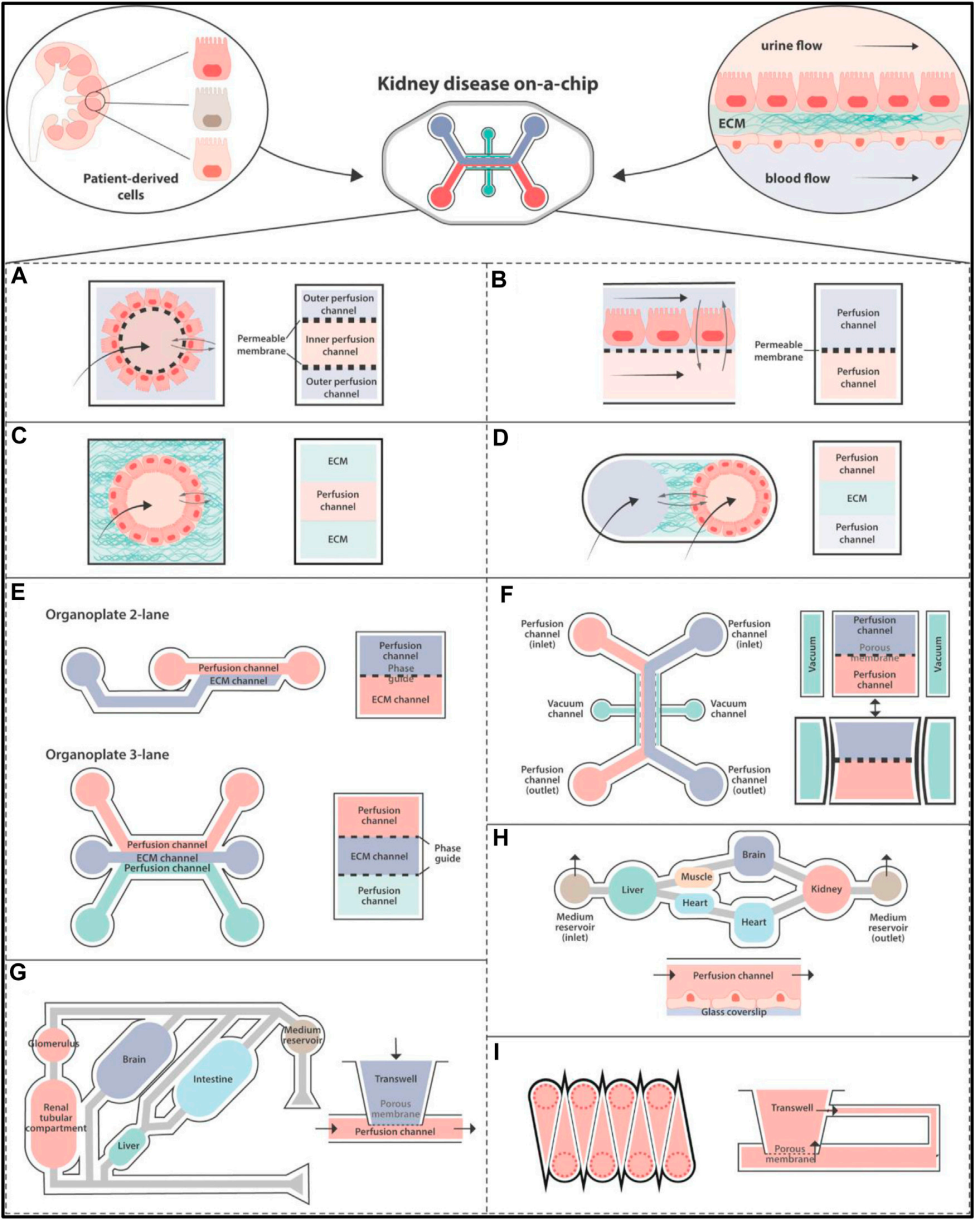
Disease Modelling in organ on chip platform: Hollow fiber in **(A)** with a cell monolayer on the outside. **(B)** A porous membrane separating multiple channels. Single **(C)** and dual **(D)** channels are enclosed in ECM-like substances. **(E–I)** Diagrams of organ on chip platform that are currently on the market that have been used to create kidneys models. **(E)** MIMETAS BV offers a number of models, such as the Organoplate 2-lane with dual channels and the Organoplate 3-lane 40 with three. The “PhaseGuideTM” is a meniscus-forming barrier that prevents direct cell contact with an ECM-filled channel, separating adjacent channels instead of membranes. **(F)** Emulator chips have four channels. A porous membrane separates the dual perfusable channels for cell culture, and the laterals are attached to a vacuum pump that exerts stretching pressures on the membrane. **(G)** The multi-organ-on-organ on chip platform technology used by Humimic CHIP by Tissues is based on the connection of Transwells’ porous membranes to a perfusable channel. **(H)** A continual perfusion tube is used by Hesperos to connect many wells in order to create co-cultures. **(I)** The cells are cultured in Transwells that have been altered by CN Bio Innovations. Emulate, Tissuse, and Hesperos replicate a unidirectional flow by cycling media from inlet to exit, while MIMETAS BV and CN Bio Innovations employ rocking devices to generate a bidirectional flow (Valverde et al., 2022). ECM, Extracellular Matrix.

**FIGURE 3:**
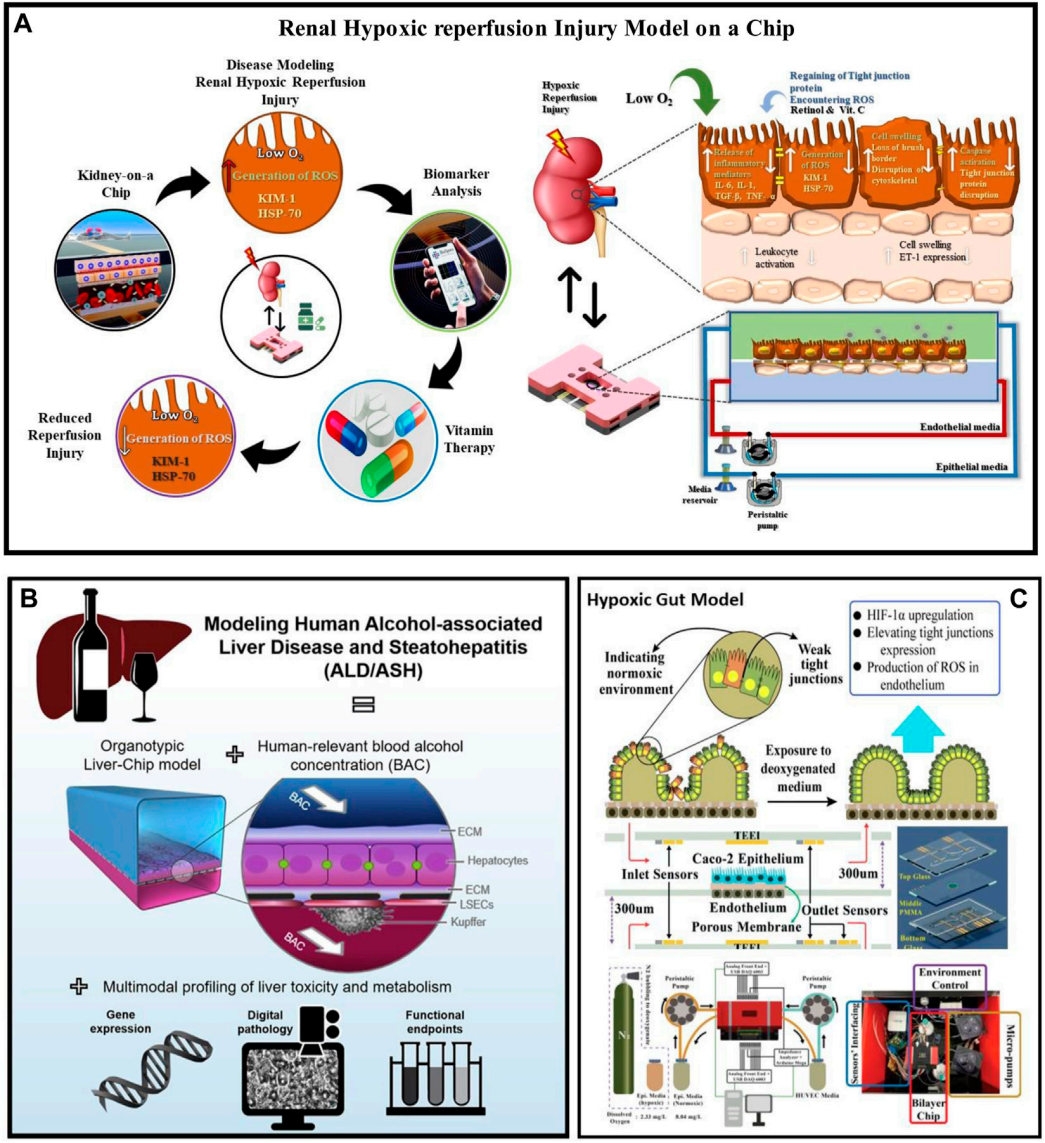
Disease condition modelling in the organ on chip platform **(A)** Renal Hypoxic Reperfusion Injury (RHR) model-on-organ on chip platform studied estimating different RHR biomarkers expression levels before and after drug administration. Additionally, treatment for RHR, retinol, ascorbic acid, and combinational dosages were investigated. RHR injury model developed by culturing hRPTECs and HUVEC on the top and bottom parts of a porous membrane. **(B)** Liver Disease Modelling in organ on chip platform: Using human-related blood alcohol concentrations (BACs) and multimodal profiling of clinically important endpoints, Liver-On-organ on chip platform modeled ALD by tri-culturing biomimetic hepatic sinusoids and bile canaliculi (Nawroth et al., 2021). **(C)** Gut Disease Modelling in organ on chip platform: By exposing Caco-2 cells in the epithelial channel and endothelial cells in the endothelial channel to deoxygenated media in the gut-on-organ on chip platform model, hypoxic condition was mimicked and monitored the gut hypoxic condition through embedded sensors (DO, ROS and TEER) (Salih et al., 2020; Khalid et al., 2022). ASH, Alcohol Related Steatohepatitis; ALD, Alcoholic liver disease; HIF-1α, Hypoxia-inducible factor 1-alpha; DO, Dissolved oxygen; ROS, reactive oxygen species; TEER, transepithelial electric resistance.

**FIGURE 4:**
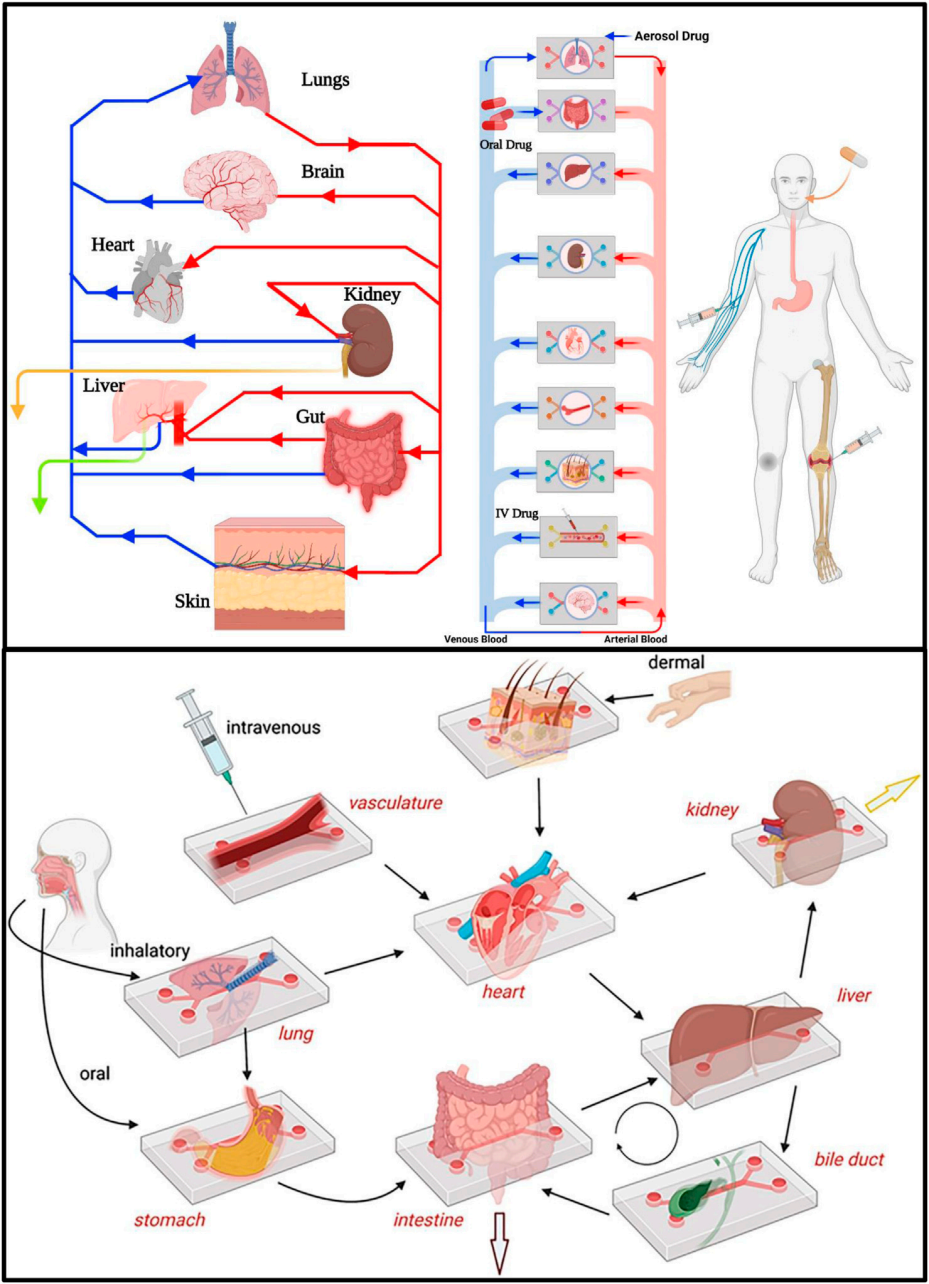
PK-PD drug Absorption, Distribution, Metabolism and Excretion (ADME) can be studied and modeled in multiorgan-on-organ on chip platform by interlinking different organs in human body via microfluidic flow channels thus mimicking human body physiology. Drug metabolism and excretion processess can be investigated by interconnected liver-kidney-on-organ on chip platform as well as with other organ’s-on-organ on chip platform models. Drug intake via modes of drug administration such as IV, oral and aerosol can be demonstrated by introducing drugs through vascular channel, lumen of an intestine-on-organ on chip platform and air space of a lung-on-organ on chip platform, respectively (van Berlo et al., 2021).

To completely understand tissue creation, function, and pathology, researchers must look into how cells in the body communicate as elements of live organs made of numerous tissue types with highly varied 3-dimensional forms, mechanical characteristics, and biochemical environment (Blin et al., 2016; Beaurivage et al., 2020; Wang et al., 2021). Most of the research on cell and tissue regulation relies on 2-dimensional cell culture models that do not replicate *in vivo* environment and thus do not have differential capacity od *in vivo* models. To overcome these limitations, 3-dimensional cell-culture models containing ECM gels have been constructed. (Han et al., 2014; Cunningham and Duester, 2015; Schuster et al., 2020; Manafi et al., 2021). This method enhances tissue structure and stimulates specific activities. Tissue-tissue interfaces, oxygen gradients, and the mechanically active tissue/organ microenvironment are examples of these. Because these cells/tissues flourish in the gel’s core, measuring physiologic diffusion gradients (for example, kidney ion transport) (Figure 2) and polarized cellular products sampling is difficult (e.g., liver bile flow) (Caballero et al., 2017; Kim et al., 2021b; Jahagirdar et al., 2021; Yoon et al., 2021). Human drug testing outcomes are commonly unstable, as evidenced by the pharmaceutical industry. More realistic microstructure, dynamic mechanical characteristics, and metabolic capabilities of organ on chip platform. These “organ on chip platform” study, human mimicking pathophysiological condition in organ-specific functions, environments and generate specific 3D complex *in vitro* disease models using microfluidics and microfabrication techniques (Figure 2; Figure 3 and Figure 4) (Sontheimer-Phelps et al., 2019; Herland et al., 2020; Chen et al., 2021b; Dér, 2021; Kang et al., 2021). The rapid development of microscale culture system designs is emerging these days that improvise the utilization of tissue and organ functions on another level. This is mainly because of the progressive advances in the fields of stem cell biology, tissue engineering, and microsystems engineering. The development of static 3D culture systems with higher structural complexity and microfluidic 3D culture devices known as organ chips incorporating dynamic fluid flow are the two main approaches to microphysiological systems development that paved the way for advanced biological mimicry (Ingber, 2022).

### Disease Modelling and parameters in organ on chip platform

Lung disease modeling: Organ-on-chip technology has emerged as a promising platform for modeling various diseases and testing drugs *in vitro*. One area where this technology is gaining attraction is in the field of lung disease modeling. By creating microdevices that mimic the structure and function of the lung, researchers can better understand the underlying mechanisms of lung diseases and develop more effective treatments. In this paragraph, we will explore several studies that have utilized organ-on-chip technology to model different lung diseases, such as pulmonary edema, lung cancer, COVID-19, asthma-COPD, and cystic fibrosis. These studies have demonstrated the feasibility of creating human disease-on-a-chip models that replicate the complex microenvironment of the lung and provide valuable insights into disease progression and drug efficacy. Huh et al. developed an organ on chip platform to study pulmonary edema, a lung disease characterized by accumulation of fluid in the lungs. The platform consisted of a PDMS device with two channels and a microporous membrane, with HPAEpic cells in the apical part and human pulmonary microvascular endothelial cells in the basal part. The flow rate for the epithelial and endothelial channels was 50 μl/h, with a shear stress of 0.2 dyne/cm2. Interleukin-2 was used to induce pulmonary edema, and angiopoietin-1 was identified as a biomarker in this study. This platform provides a physiologically relevant model for studying the mechanisms of pulmonary edema and testing potential therapeutics (Huh et al., 2012). In their study (Hassell et al., 2017), Hassell *et al.* utilized an organ-on-chip platform to generate a 3D *in vitro* model of non-small cell lung cancer (NSCLC) that mimics the organ-specific microenvironment and allows for testing of tyrosine kinase inhibitor (TKI) responses *in vivo*. The platform consisted of HPAEpic, human primary airway epithelial cells, human lung microvascular endothelial cells (HLMECs), and cancer cells. The flow rate through both endothelial and epithelial channels was set at 60 μL hr−1, and the PDMS device had two channels with a microporous membrane. The biomarkers discovered in the study were IL-6, IL-8, and VEGF. The study highlights the potential of organ-on-chip platforms in studying NSCLC and testing the efficacy of targeted therapies (Kim et al., 2020; Zhang et al., 2021a) In a recent study by Jungwook et al., an organ on chip platform was utilized to investigate the efficacy, vascular network, and cancer toxicity of Paclitaxel, a chemotherapeutic drug commonly used to treat advanced stages of lung cancer. The platform consisted of a three-chambered PDMS microfluidic chip lined with primary human umbilical vein endothelial cells (HUVECs), adenocarcinoma cells, epithelial cells, and lung fibroblasts. Perilipin-1 and Leptin were identified as biomarkers in this study. The flow rate of endothelial and epithelial channels was carefully controlled at 70 μl/h to optimize experimental conditions (Benam et al., 2016b; Amin et al., 2017). In their study, Jain *et al.* developed a lung alveolus chip by modifying a human lung on a chip platform. They achieved this by culturing HLMECs and HPAEpic in a multi-channeled PDMS device and perfusing the device with whole blood instead of cell culture media through the microfluidic channels. The flow rate of the media through the endothelial channel ranged from 275 to 750 s-1. Additionally, they identified protease activated receptor-1 (PAR-1) as the biomarker for their study (Benam et al., 2016b). Zhang et al. developed a human lung alveolus on a chip infected with SARS-CoV-2, which contained HPAEpic, peripheral blood mononuclear cells, and HLMECs cultured in a multi-channel PDMS organ on chip platform. This study mimicked SARS-CoV-2 infection and the associated immunological responses to lung injury *in vitro*. The flow rate in both the endothelial and epithelial channels was reported to be 50 μl/h. ACE2, TMPRSS2, IL-16, IL-11, CXCL11, CCL15-CCL14, CCL15, and CCL23 were the biomarkers discovered during the study (Zhang et al., 2021a). Kambez *et al.* conducted a study on an organ-on-chip platform to model asthma-COPD disease. The study induced IL-13 to the lung epithelial region to examine asthmatic conditions and bacterial and viral infections. The platform was also utilized for lung inflammatory disorder modeling, identifying new biomarkers, analyzing drug efficacy, and cytokine secretion from both epithelial and endothelial regions. RANTES, IL-6, IP-10, and M-CSF biomarkers were analyzed, and the model featured HLMECs in the basal part and HPAEpic in the apical part of the chip. The flow rate for both channels was 60 μl h−1, and the shear stresses were 1 dyn cm2 (Benam et al., 2016a). Roberto et al. successfully replicated a cystic fibrosis (CF) model using an organ-on-chip platform. The study utilized cells from CF patients cultured in a multi-channeled chip to analyze inflammation, bacterial infection, mucus secretion, and *P. aeruginosa* growth in a CF lung airway chip. Primary human lung microvascular endothelial cells (HMVEC-L) and human bronchial epithelial cells (HBEC) collected from CF patients were used. The flow rate was set at 45 μL/h in both epithelial and endothelial channels of the multi-channeled organ-on-chip platform. Moreover, the study examined the expression of biomarkers IL-8, IP-10, GM-CSF, MIP1-alpha, and IL-6 (Plebani et al., 2022). Kambez *et al.* used an organ-on-chip platform to model human small airways and study lung injury resulting from tobacco smoking. The researchers connected e-cigarettes and traditional smoking devices to the airway organ on a chip platform that contained cultured healthy and COPD patient HBEC and primary human airway epithelial cells (hAECs) in an air-liquid interface. During the study, the expression of various genes such as SPRR3, IL-8, NRCAM, MT1H, ATP6V0D2, TMPRSS11E and TMPRSS11F, MMP1, RPTN, ANKRD22, and TSPAN7 were detected (Ahmed et al., 2022d). Longlong et al. investigated the impact of coronavirus-2 and influenza A infections on the bronchial airway using an organ-on-chip platform. They administered oseltamivir with nafamostat to the infected lung organ-on-chip platform to assess its efficacy, immune response, and cytokine production. The study also evaluated the effectiveness of antimalarial drugs, amodiaquine and hydroxychloroquine, in a SARS-COV-2 infected model. A two-channel PDMS microfluidic device was used to model the disease with a flow rate of 60 μL/h, and the expression of biomarkers, including IFN-β, IP-10, Interleukin-6, RANTES, and MCP, were analyzed (Gard et al., 2021). In their recent study, Gard *et al.* presented a novel 96-device platform that enables the investigation of COVID-19 and Influenza A virus infections. This platform is specifically designed for the culture of human pulmonary alveolar epithelial cells (HPAEpic) and human bronchial epithelial cells (HPBECs) in an air-liquid interface. The authors demonstrated the real-time and high-throughput sensing capabilities of the platform for the analysis of viral infection and drug efficacy. The multi-channel plastic microfluidic device utilized in this platform features a 1 μL/min flow rate in the epithelial channel (Gard et al., 2021). Gard et al. further analyzed the expression of biomarkers TMPRSS2 and ACE2 (Gard et al., 2021; Ahmed et al., 2023). Janna *et al.* developed a multi-channel PDMS lung airway organ-on-chip platform that was lined with rhinovirus-infected airway epithelial cells to replicate viral-stimulated asthma exacerbation and neutrophil transmigration through the lung airway epithelium. The platform was designed to mimic the human lung airway, with a shear stress of 1 dyn/cm2 and a flow rate of 60 μL/h in both epithelial and endothelial channels. The study identified altered IFN-1, CXCL10, and IL-6 biomarkers. This platform provides a valuable tool to investigate the underlying mechanisms of asthma and viral infection (Nawroth et al., 2020; Ahmed et al., 2022e). Longlong *et al.* discovered a novel class of immune response-stimulating RNAs while investigating host genes linked to influenza infection in human lung epithelial cells using siRNAs. They utilized a PDMS-based organ-on-chip platform device with a microporous membrane between the two channels and measured interferonβ expression. The platform was designed to replicate the human lung airway, with a flow rate of 60 μL/h in both endothelial and epithelial channels. These findings suggest that the organ-on-chip platform can be a powerful tool for studying the molecular mechanisms of immune response to viral infections in human lung epithelial cells (Si et al., 2021b). Overall, lung-on-chip technology holds great promise for improving our understanding of lung function and disease, and for developing more effective treatments for lung-related illnesses.

Blood-Brain-Barrier (BBB), nerve, and blood vessel disease modeling: The blood-brain barrier (BBB) is a highly specialized interface between the brain and the blood that protects the brain from harmful substances. Modeling BBB diseases on organ-on-chip platforms allows for the study of disease mechanisms and the development of new treatments in a more physiologically relevant environment. Vatine *et al.* developed a BBB-on-organ on chip that recapitulates the human BBB and can forecast individual variability. This model was composed of neurons, human brain astrocytes (HBA), human brain vascular pericytes (HBVP), and iPSC- Brain microvascular endothelial cells (iPSC-BMEC), and utilized a PDMS based multi-channel device with a microporous membrane. The study also assessed the expression of biomarkers GFAP, a-SMA, and PDGFRb under different shear stress conditions, providing insight into the BBB’s response to physical stimuli (Vatine et al., 2019). To investigate the infection and infiltration of the BBB by living microorganisms, Kim *et al.* developed a functional BBB recapitulating cerebro-vascular unit on organ on chip. This model was co-cultured with HBVP, BMEC, and Human neural stem cells (HNSCs), and examined the expression of various biomarkers such as TIMP-1, PTX3, TSP-1, SERPINE1, and ET-1. The study’s findings have important implications for understanding and treating neurological diseases caused by infection (Kim et al., 2021b). Losif *et al.* focused on modeling the Substantia Nigra on a brain organ on chip to study the pathological process of Parkinson’s Disease (PD). This model was composed of dopaminergic neurons, BMECs, HBA, human microglia, and human brain pericytes under controlled fluid flow. The study investigated the expression of COL3A1, CENPE, KIF15, and SERPINA1 biomarkers, providing insights into the molecular mechanisms underlying PD (Pediaditakis et al., 2021). Spijkers *et al.* have presented a method for developing a 3D compartmentalized neurite outgrowth model on a high-throughput, biocompatible, and non-absorbing 3-channeled plastic organ-on-chip platform. The study used iPSC-derived motor neuron and progenitor cells, which were cultured in the platform to assess the impact of physiologically relevant toxic substances such as sodium arsenate and glutamate, which induce neuronal diseases. The model was validated by analyzing the expression of biomarkers such as islet-1 (ISL1), CHAT, NFH, SLC18A3, choline acetyltransferase (CHAT), VACHT, S100, SMI32 MAP2, and TAU (MAPT)/TAU. The platform offers a novel approach for the development of a reliable and high-throughput method to screen toxic substances and evaluate their effects on neuronal cells, which could be beneficial for drug discovery and neurodegenerative disease research (Spijkers et al., 2021). Ribas *et al.* developed a PDMS-based organ on chip platform to model progeria and investigate the impact of biomechanical strain on vascular disease and aging. The study induced vascular damage using pathophysiological strain on iPSC-derived smooth muscle cells, followed by angiotensin II treatment. The platform was validated through the expression of biomarkers CAV1, SOD1, NADPH, IL6, IL1B, and JUN (Ribas et al., 2017). Cho *et al.* proposed a pathophysiologically relevant lymph angiogenesis organ on chip platform model to investigate the response of a human-mimicking lymphatic vessel system to biochemical and physical features. The study assessed the role of the tumor microenvironment (TME), extracellular matrix (ECM), tumor mass, angiogenesis factors, and metastasis. The study used human dermal lymphatic endothelial cells and breast cancer cells lined in a three-channeled PDMS microfluidic device containing ECM gel. The given flow rate range in the endothelial channel was 0.49 to 0.09 m/s. The study also identified the circulating biomarkers CCL21 and CCR7 (Cho et al., 2021). Hachey *et al.* demonstrated a microvascularized colorectal cancer model created in a 3-channeled PDMS organ on chip platform device, which was lined with primary lung fibroblast, colorectal cancer cell, and human endothelial colony forming cell-derived endothelial cell with extracellular matrix. This study evaluated real-time drug response in colon tumor, microvascularized tumor environment response, gene expressions, and stromal-tumor interactions. Validation of this model was achieved through biomarker expression, including ANGPT2, NID2, APLN, CYTL1, DLL4, and IGFBP4 (Hachey et al., 2021). Chou *et al.* demonstrated the culturing, maturation, and differentiation of immune cells (CD34^+^) in a physiologically relevant vascularized bone marrow model on an organ-on-chip platform. In this study, a PDMS-based device was used, which consisted of a layer of HUVEC on the basal part, and bone marrow stromal cell, mononuclear cells, and hematopoietic CD34^+^ cell on the apical region of the chip. The primary focus of this study was to investigate the impact of ionizing radiation therapy and chemotherapeutic drug exposure on bone marrow injury and recovery. The researchers also created Shwachman diamond syndrome on this physiologically relevant bone marrow organ-on-chip platform and found an abnormality in neutrophil maturation due to hematopoietic defects. This study validated the model through the expression of various biomarkers, including CD34, CXCL12, VEGF, MMP9, and FGF-2 (Chou et al., 2020). In their study, Cho *et al.* developed a physiologically relevant artery model on a multichannel PDMS-based organ-on-chip platform to investigate inflammation and stenosis. The model was created through co-culturing of human umbilical vein endothelial cells (HUVECs) and smooth muscle cells, followed by inducing physiochemical factors to create inflammation and stenosis at a flow rate of 795 μL/min. The artery model was induced by varying concentrations of TNF-α, and the expression of inflammation biomarkers ICAM-1 and vWF was analyzed, while the THP-1 adhesion assay was used to evaluate stenosis (Cho and Park, 2021). Barrile *et al.* developed a micro-engineered vessel on an organ-on-chip platform, which was lined with HUVEC cells and infused with human blood to investigate crucial aspects of thrombosis. The flow rate of the platform was maintained at 60 μl/min. The study aimed to evaluate platelet aggregation and adhesion, endothelial activation, thrombin anti-thrombin complexes, and fibrin clot formation in the chip-effluent in response to Hu5c8 and in the presence of soluble CD40L. This model was validated through the expression of biomarkers such as Thrombin antithrombin complex (TAT), plasminogen activator inhibitor-1 (PAI-1), and SERPINE-2 (Barrile et al., 2018).

Mammary gland, cartilage, eye and heart disease modeling: Matthew *et al.* developed an organ-on-chip platform to study the coordinated morphogenic interplay between the epithelium and vasculature in the context of breast cancer. This platform comprises a multi-channel PDMS-based device with a microporous membrane separating two channels, one containing a mammary duct and the other a perfused endothelial vessel. Human mammary epithelial cells (HMECs) and human dermal microvascular endothelial cells (HDMECs) were utilized to represent the breast cancer microenvironment. The model assessed the amplification of HER2/ERBB2 and activation of PIK3CA(H1047R) alteration, and evaluated the production of the IL-6 biomarker in response to PI3K-H1047R-related vascular dysfunction. The model was subjected to a shear stress of 3.3 Dyn/cm2. This study provides insight into the coordinated interplay between the vasculature and epithelium in breast cancer, which can aid in the development of effective cancer therapies (Kutys et al., 2020). Mondadori *et al.* developed a microfluidic organotypic model to investigate the extravasation of monocytes, which are the precursors of infiltrating macrophages in the context of synovial and cartilage compartments. The model employed synovial fibroblasts, human primary monocytes, articular chondrocytes, and HUVECs in a two-channel PDMS microfluidic device with a range of endothelial channel flow rates (5, 10, 15, or 30 l h1) and 1–5 dyne cm2 shear stress. The study aimed to examine the biomarkers ICAM-1 and VCAM-1 in response to different flow rates. This organotypic model provides a valuable tool to investigate the role of monocyte extravasation in the pathophysiology of inflammatory diseases such as rheumatoid arthritis (Mondadori et al., 2021). Achberger et al. developed a microphysiologically relevant retina-on-a-chip platform model using human induced pluripotent stem cell-derived retinal cells, including ganglion cells, bipolar cells, horizontal cells, amacrine cells, Müller glia, and photoreceptors. This study aimed to evaluate human-like phagocytosis in the outer segment and calcium dynamics of the retina. The multi-lined PDMS-based chip had a microporous membrane and was cultured with retinal cells on both the apical and basal sides at a flow rate of 20 μl/h. Additionally, this study assessed the retinopathic side effects of chloroquine anti-malarial and gentamycin antibiotic drugs by examining the expression of PNA, lectin, PAX6, MITF, melanoma gp100, Ezrin, and VEGF-A biomarkers (Achberger et al., 2019). Chen et al. developed a 3D microenvironment model of heart valves and blood vessels in a double-channel PDMS-based microfluidic device. They cultured primary porcine aortic valvular interstitial cells in hydrogel to mimic the valvular or vascular microenvironment. The study employed a shear stress of 20 dynes/cm2 or 0 dynes/cm2 with a flow rate of 1.3 μL/min in the epithelial channel. The researchers improved upon their previous work by integrating a cell-laden 20 hydrogel to better replicate the microenvironment. The study found that pathological differentiation of valvular interstitial cells to α-SMA-expressing myofibroblasts was suppressed in the presence of valvular endothelial cells, indicating the potential of this model to explore pharmacological therapies for valvular disease. This model may help to advance our understanding of valvular disease and provide new insights for drug discovery (Chen et al., 2013; Meghani et al., 2017). Liu *et al.* conducted a study to investigate the effect of acute hypoxia on cardiac function by utilizing a newly developed bioelectronics ischemia-on-a-chip platform model integrated with extracellular and intracellular bioelectronic devices. The study employed mouse atrial cardiomyocytes on a single channel PDMS device and induced hypoxia by exposing them to 1% O2 media. The device had a flow rate of 40 μL/h in the epithelial channel. The nanopillar approach used in this study enabled precise action potential measurements, action potential measurements at the single-cell level, and mapping of intracellular characteristics within the same sample spatially. The key biomarker identified in this study was HIF-1∝. The results of this study can have significant implications in understanding the effects of hypoxia on cardiac function and the development of potential therapeutic interventions (Liu et al., 2020).

Renal disease modeling: Zhou et al. developed a glomerulus-on-a-chip platform to mimic human hypersensitive nephropathy, a pathophysiological condition of the kidney. This study investigated the effect of fluidic flow and shear stress on the glomerular microenvironment, including cell tight junction damage, podocyte and endothelial cell injury, and leakage. The two-channel PDMS-based organ-on-chip platform contained cultured human renal glomerular endothelial cells (HRGECs) and podocytes. The model was validated using biomarkers such as F-actin, CD-31, and synaptopodin (Zhou et al., 2016). Salih *et al.* developed a renal hypoxic reperfusion (RHR) injury-on-a-chip platform model to investigate the role of antioxidant vitamins in preventing RHR injury. This model mimicked the pathophysiological relevance of RHR injury by culturing human umbilical vein endothelial cells (HUVECs) on the basal part of a microporous membrane and renal proximal tubular epithelial cells (RPTECs) on the apical part of the membrane. A TEER sensor was used to analyze the RHR injury throughout the experiment. The authors tested combinational and individual doses of retinol and ascorbic acid and found that combinational vitamin therapy can reduce the risk of RHR injury. The flow rate in the proximal tubule part was 25 μl/min and in the endothelial region was 45 μl/min with fluidic shear stress of 0.2 dyn/cm2. They also examined the secretion of several biomarkers, including KIM-1, HSP70, IL-6, and ET-. This study provides insights into developing a treatment for RHR injury (Chethikkattuveli Salih et al., 2022). Naik and colleagues developed a renal proximal tubule-on-a-chip platform model to investigate Lowe syndrome/Dent II disease. The model was created using a multi-channeled plastic microfluidic device with an ECM gel to create a 3-dimensional proximal tubular design. The fluidic flow was specifically controlled with a shear stress range of 0.06–0.3 dyn/cm2. In this study, the expression levels of biomarkers SNAI2, TAGLN, COL1A1, MMP1, and COL5A1 were measured to validate the drug target. This platform model provides a promising approach to study the pathophysiology of Lowe syndrome/Dent II disease, as well as to develop and test potential treatments (Naik et al., 2021). Wang *et al.* developed an *in vivo* mimicking distal tubule on organ on chip platform model to study sodium reabsorption and barrier structure. The focus of the study was to investigate the impact of Pseudorabies Virus (PrV) infection on sodium reabsorption, transportation, microvilli transformation, barrier breakage, and regulation of electrolyte in the distal tubule. The platform model utilized a multi-channeled PDMS chip separated by a nanoporous membrane and lined with Madin Darby Canine Kidney cells cultured at a fluid flow rate of 11.3 μL/h. The study validated the model by examining the expression of different biomarkers, including sodium, potassium, ATPase, and ZO-1 (Wang et al., 2019). Lin *et al.* have developed a 3D vascularized renal proximal tubule model, which allows for human physiologically relevant renal reabsorption, including glucose reabsorption and albumin uptake. This study evaluated cellular injury, dysfunction, electrolyte transportation under hyperglycemic conditions, and potential treatment options such as Dapagliflozin and glucose inhibitors. The model consisted of a permeable extracellular matrix with proximal tubule epithelium and microvascular endothelial cells cultured at a flow rate of 3 μL/min and a shear stress of 0.3 dynes/cm2 in both channels. Furthermore, the proximal tubule organ-on-chip platform was validated through various biomarkers including sodium, potassium, ATPase, actin, CD31, laminin, Ki-67, and SGLT2 (Lin et al., 2019). This models provides an efficient and accurate platform for studying kidney disease pathogenesis and potential treatments.

Gut disease modeling: Rogal *et al.* developed an organ on chip platform to investigate fully developed human white adipocytes. The multilayer device, called White Adipose Tissue (WAT)-on-a-chip, was designed with tissue chambers to maintain 3D tissues based on primary human adipocytes. Perfusion of nutrition was provided through medium channels. The study utilized a two-channel PDMS system separated by a microporous membrane with a flow rate ranging from 20 to 40 l/h. The expression of lactate dehydrogenase was also examined in this research to evaluate cellular damage. The WAT-on-a-chip platform has significant potential in various areas of research, including obesity, drug screening, and metabolism (Rogal et al., 2020). Apostolou *et al.* developed a colon-intestine on an organ on chip platform with human colonic microvascular endothelial cells and colon crypt-derived epithelial cells to investigate the increased intestinal permeability in humans. The platform used a multi-channel polydimethylsiloxane (PDMS) design at a flow rate of 60 μL/h. This *ex vivo* model examined the effect of proinflammatory cytokines on the intestinal epithelial barrier and identified a novel mode of action of IL-22. Specifically, the study evaluated the impact of IL-22 on colon epithelium integrity and its ability to mimic the barrier breakdown initiated by interferon. The results demonstrated that IL-22 acts as a cytokine that breaks down the barrier by interacting with its receptors encoded on the colon-intestine-on-a-chip platform. The study also identified several biomarkers, including Alpi, STAT3, Bmi1, Na+K+ ATPase, Muc2, SLC26A3, caspase 3, 18S, ChgA, interleukin 22, and Lgr5, to validate the model’s effectiveness (Bein et al., 2022). Strelez et al. developed a colorectal cancer (CRC) on organ-on-chip platform model that simulates the human physiological structure and microenvironment of the colorectal tissue to investigate tumor intravasation. Their study utilized a PDMS-based multi-channeled CRC-on-a-chip platform with a basal channel lined with HUVECs and an apical channel lined with intestinal epithelial cells (IEC), separated by a porous membrane with integrated fluid flow and cyclic stretching to mimic peristalsis. The flow rate provided on both channels was 30 μL/h. The study aimed to examine the cellular variability arising from CRC progression using effluent assessments and on-chip imaging. The biomarkers examined in this study were ZO-1, vimentin, and E-cadherin (Strelez et al., 2021). Guo et al. developed an intestinal infection-on-a-chip platform model to study SARS-CoV-2-generated intestinal infection and its impact on human intestinal physiology and pathology. The platform was designed using a multi-channel PDMS system that incorporated both human umbilical vein endothelial cells (HUVECs) and intestinal epithelial cells (which secrete mucin) to recreate the essential features of the vascular endothelium-intestinal epithelium barrier. The intestinal epithelium exhibited a propensity to viral infection and showed clear morphological abnormalities, including damaged intestinal villi, scattered mucus-secreting cells, and diminished E-cadherin expression, indicating that the virus had compromised the integrity of the intestinal barrier. Similarly, the adherent junctions in the vascular endothelium were disturbed, indicating aberrant cell morphology. Following viral infection, the transcriptional findings revealed unusual protein metabolism and RNA, along with triggered immune reactions, in both epithelial and endothelial cells. These findings explain the damage to the intestinal barrier that resulted in gastrointestinal issues. The study also analyzed various biomarkers including NF, interleukin-6, CXCL1, CXCL11, CXCL10, CCL5, and CSF3 (Guo et al., 2021). Bein et al. conducted a study to investigate Environmental Enteric Dysfunction (EED)-associated injury of the intestine using a multi-channel intestine-on-a-chip platform made of polydimethylsiloxane (PDMS). They cultured epithelial cells derived from EED patients or healthy human intestinal organoids on the epithelial channel under a flow rate of 60 μL/h with a culture medium that was deficient in tryptophan and niacinamide. This study revealed that the clinically identified EED phenotype is induced by both nutrient deficiencies and epigenetic or genetic modifications in the intestinal epithelium. They compared EED patient-derived samples with the human intestine-on-a-chip platform and investigated phenotypical responses to nutrient problems that are commonly observed in intestinal diseases, such as dysfunction of the barrier and blunting of villi. Furthermore, they examined the expression of biomarkers such as neuregulin-4, SMOC2, and apolipoprotein B. The study sheds light on the mechanism behind EED and provides a new tool for studying intestinal diseases (Bein et al., 2021).

Liver and Pancreatic disease modeling: Freag *et al.* investigated the development of steatosis and non-alcoholic steatohepatitis (NASH) using a liver-on-organ-on-chip platform, which was induced by palmitic acid, lipopolysaccharide (LPS), and oleic acid. The platform consisted of four key primary human liver cells, including Kupffer cells (KCs), hepatic stellate cells (HSCs), hepatocytes (HCs), and liver sinusoidal endothelial cells (LSECs), and was used to evaluate a novel NASH therapy drug, elafibranor. The researchers observed that elafibranor inhibited the development of NASH-specific hallmarks, leading to a reduction in intracellular lipid accumulation (∼8-fold), a significant decrease in HSC activation, a reduced HCs ballooning (3-fold), and a considerable reduction in inflammatory and profibrotic marker levels compared to control groups. Albumin, urea, α-SMA, COL1A1, and TIMP-1 were identified as biomarkers in this study. The study effectively recreated the physiological human liver cellular microenvironment in a multi-channeled plastic-based microfluidic device. This research provides a promising tool for the investigation of the pathogenesis of liver diseases and the development of novel treatments (Chethikkattuveli Salih et al., 2021; Vormann et al., 2022). Ehrlich *et al.* developed a novel sensor-integrated liver-on-chip array using microfluidic electrochemical sensors to simultaneously detect lactate, glucose, and temperature in real-time, and tissue-embedded microprobes with two-frequency phase modulation to measure oxygen. They utilized a single-channeled plastic device to investigate the impact of two drugs, Valproate and Stavudine, on the human liver. The study was performed at a flow rate of 2 μL/min in the epithelial (human hepatocytes) and endothelial channels (microvascular cardiac endothelial cells). The principal finding of the study emphasized the significance of monitoring metabolic stress as a reliable indicator of clinical response since a rapid disturbance of metabolic balance was observed without reaching the threshold of cell damage. Additionally, the authors observed that Stavudine exposure produced a brief increase in lipogenesis, whereas Valproate exposure resulted in a prolonged 15% rise in lipogenesis followed by mitochondrial stress, indicating a disruption in β-oxidation. Biomarkers CPT1, COX2, UPC2, and CYP2E1 were identified in this study (Ehrlich et al., 2018). Ortega-Prieto *et al.* developed a 3D microfluidic liver-on-a-chip platform system, tolerant to Hepatitis B Virus (HBV) infection, that can be maintained for up to 40 days. In this study, primary human hepatocytes (PHH) and Kupffer cells (KCs) were cultured on a single-channeled plastic platform, and infected with infectious recombinant HBV. The flow rate of the epithelial and endothelial channels was 1.0 μL/s. The main biomarkers recognized in this study were IL-6 and TNF-α. This platform provides an excellent model to study complex host/pathogen diseases, such as the interaction between HBV and PHH, even with low-titer patient-derived HBV. The results demonstrate the potential of this system to investigate the mechanisms underlying HBV infection and to test novel therapies (Ortega-Prieto et al., 2018). Glieberman et al. introduced a single-channel thermoplastic-based Islet-on-a-chip platform that allows for scalable design, automated islet loading and stimulation, and insulin measurement. The study utilized human cadaveric islets and stem cell-derived β (SC-β) cells in a sheer stress environment of 0.3 dynes/cm2. To induce a diabetes-like condition, a mixture of high and low concentrations of insulin or glucose was administered. The flow rate of the epithelial cells was maintained at 1.6 μL min−1. This platform provides a scalable and versatile tool for diabetes research with advanced microfluidic technologies that enable testing of Islets (Glieberman et al., 2019).

Lymphatic vessel disease modeling: The development of organs-on-chip has led to significant advances in pharmacological and toxicological research. However, there has been a recent recognition of the need to develop lymph node-on-chip models due to the crucial role in maintaining fluid homeostasis and immune surveillance in the body. Likewise, lymphatic vessels, which transport immune cells and antigens to lymph nodes, have been identified as important components for integration into organs-on-chip models. Developing biomimetic lymphatic vessels-on-chip models may facilitate the investigation of immune cell trafficking and drug candidate interactions. These models could also enable high-throughput screening of drugs and nanoparticles for their effects on lymphatic vessels (Choi and Meghani, 2016; Shanti et al., 2021). Fathi et al. developed a microfluidic device to model lymphatic vessel behavior in both healthy and diseased states using primary human lymphatic endothelial cells. The study simulated healthy conditions by applying cyclical shear of 0.092 Pa (0.92 dyn/cm2) to the cells, and disease conditions were simulated by either removing all flow from the culture or by applying cyclical high shear (0.67 Pa, 6.7 dyn/cm2) to the cultures. The developed device and rotating platform provide a versatile *in vitro* system for evaluating lymphatic behavior under physiological shear flows. The cell count per unit area increased with the application of shear flow, promoting cell proliferation, while static conditions led to higher TNF-α and IL-8 levels per cell. This study is the first pumpless microfluidic model of lymphatic vessels that evaluates the effects of healthy and diseased flow conditions on primary human lymphatic endothelial cells, and it demonstrates the potential of this microfluidic device for investigating lymphatic vessel behavior and interactions with other cells such as cancer cells, T cells, or dendritic cells (Fathi et al., 2020). In another study, In *et al.* created a microfluidic valvular chip using polydimethylsiloxane (PDMS) to investigate the mechanics of water transport inside lymphatic vessels. The chip consisted of a central channel and multiple side channels separated by a thin wall. They also developed a numerical model to study the flow characteristics observed in the chip, allowing for a more thorough parametric study than using the chip alone. Parameters such as the viscosity and contraction fraction were varied to determine the optimum period for maximizing water transport. Additionally, the authors examined the mechano-temporal correlations between adjacent valves and the transmissions of hydrodynamic forces to regulate water transport. Ns, or the number of chambers simultaneously compressed, and Nr, or the number of retrograde orders for compressing two successive chambers, were found to play a significant role in promoting water transport downstream. Although the microfluidic valvular chip and numerical model were over-simplifications of a lymphatic vessel *in vivo*, the chip demonstrated technical advances in enabling discrete unidirectional movement of fluid in the picoliter range and can potentially be used for *in vitro* mechanobiology studies of endothelial cells (Ryu et al., 2021). Lymph node-on-chip models have the potential to not only accelerate drug and vaccine development but also pave the way for personalized medicine. Incorporating patient-derived immune cells into lymph node-on-chip devices can lead to a more precise and effective treatment of different diseases. For instance, resistance to cancer therapy can be addressed by studying the specific cellular response to cancer therapy using patient-derived cells in a physiologically relevant microenvironment. Future lymph node-on-chip designs should incorporate multiple design elements to create a complete, multifunctional lymph node that is biologically sustainable. Microfluidic pathways, 3D cellular matrix, co-culture of multiple cell types, chemotaxis, and cellular communication can make future lymph node devices more biologically relevant. These models will allow investigations into different immune cellular events and their association with drug discovery and vaccine development. For example, they could be used for examining effects of pharmaceutical drugs on immune cell motility, antigen presentation, T cell activation, B cell differentiation, and antibody production in a more realistic 3D scenario. However, it is crucial to validate lymph nodes-on-chip against both animal and clinical trial results to determine the reliability of these models as predictive tools. Easy-to-use, relatively inexpensive, and highly reproducible lymph node-on-chip platforms are necessary for widespread adoption (Meghani et al., 2018; Shanti et al., 2021; Samantasinghar et al., 2023). (Different organs, its associated diseases and the disease modeling parameters in organ on chip platform have been summarized in Table 1).

**TABLE 1:** Organ, Disease and Disease Model Parameters in organ on chip platform.

Organ	Disease condition	Cells used	Platform and materials	Shear stress	Flow rate		Inducer	Biomarker	Biochemical assays	Clinical relevance	References
					Epithelial channel	Endothelial channel					
Lung alveolus	Pulmonary edema	Human pulmonary microvascular endothelial cells, alveolar epithelial cells	PDMS 2-Channel Microporous	∼0.2 dyne/cm2	50 μl/h	50 μl/h	IL-2	Angiopoietin-1 (Ang-1)	Permeability assay and immunostaining	Drug toxicity	Huh et al. (2012)
Lung alveolus	Non-small cell lung cancer	Human primary airway epithelial cells, primary human primary alveolar, epithelial cells, human lung microvascular endothelial cells, cancer cells	PDMS 2-Channel Microporous	nil	60 μL/h	60 μL/h	TKI rociletinib	IL-6, IL-8, and VEGF	Cytokine analysis, EGFR phosphorylation analysis, immunostaining, and histological staining	Site-specific and mechanosensitive tumor growth, drug efficacy	Hassell et al. (2018)
Lung alveolus	Lung cancer	primary human umbilical vein, endothelial cells, epithelial cells, cancer cells, human lung fibroblasts	PDMS 3-Channel ECM gel	nil	70 μl/h	70 μl/h	paclitaxel BM-1	perilipin-1 and Leptin	Human leptin ELISA kit, T lymphocytes adhesion assay, liposome binding assay and immunostaining	Drug efficacy	Paek et al. (2019)
Lung alveolus	Thrombosis	primary human alveolar epithelial primary human microvascular endothelial cells	PDMS 2-Channel Microporous	3 N m-2			LPS endotoxin TNF-α	protease activated receptor-1 (PAR-1)	Permeability assays and immunostaining	Drug efficacy	Jain et al. (2018)
Lung alveolus	Virus infection (SARS-CoV-2), inflammation	human alveolar epithelial cells, pulmonary microvascular endothelial cells, human peripheral blood mononuclear cells	PDMS 2-Channel Microporous	nil	50 μl/h	50 μl/h	SARS-CoV-2 virus	ACE2, TMPRSS2, IL-16, IL-11, CXCL11,CCL15-CCL14, CCL15 and CCL23	Permeability assay, western blot analysis, analysis of inflammatory cytokines, immunostaining, qRT-PCR, and RNA-seq	Drug efficacy	Zhang et al. (2021a)
Lung airway	Asthma, COPD	primary human airway epithelial cells, human lung microvascular endothelial cells, immune cells	PDMS 2-Channel Nanoporous	1 dyn cm−2	60 μl/h	60 μl/h	viral mimic poly or with lipopolysaccharide endotoxin	RANTES, IL-6, IP-10, and M-CSF	Cytokine assay, adhesion assay, flow cytometry analysis, immunostaining and RNA-seq	Inflammation, COPD exacerbations, drug efficacy	Benam et al. (2016a)
Lung airway	Cystic fibrosis, inflammation, bacterial infection	primary human lung microvascular endothelial cells, human bronchial epithelial cells, Immune cells, Bacteria	PDMS 2-Channel Microporous	nil	45 μL/h	45 μL/h	*P. aeruginosa* bacteria	IL-8, IP-10, GM-CSF, MIP1-alpha, and IL-6	Cytokine assay and immunostaining	Hyperinflammation in cystic fibrosis	Plebani et al. (2022)
Lung airway	COPD exacerbation by smoke inhalation	Primary human small airway epithelial cells	PDMS 2-Channel Nanoporous	nil	nil	nil	Cigarette smoke	IL-8, MT1H, TMPRSS11E and TMPRSS11F, MMP1, SPRR3, RPTN, ATP6V0D2, ANKRD22, TSPAN7, and NRCAM	Analysis of chemokines and cytokines, qRT-PCR, western blot analysis and microarray analysis	Replication of clinical phenotype	Benam et al. (2016b)
Lung airway	Virus infection (influenza, pseudotyped SARS-CoV-2), inflammation	Primary human lung bronchial airway epithelial basal stem cells, primary human pulmonary microvascular endothelial cells, immune cell	PDMS 2-Channel Microporous	nil	60 μL/h	60 μL/h	influenza A virus	IL-6, IP-10, RANTES, IFN-β, and MCP-1	Permeability assay, plaque-formation assay, analysis of cytokines and chemokines, infection assay, native SARS-CoV-2 *in vitro* infection assay, immunostaining, and RT–qPCR	Drug repurposing, drug efficacy	Si et al. (2021a)
Lung airway	Virus infection (SARS-CoV-2, HCoV-NL63, influenza), inflammation	normal human bronchial epithelial cells	Plastic 2-Channel Nanoporous	nil	1 μL/min	nil	infuenza A virus and coronavirus infections	TMPRSS2 and ACE2	Immunostaining and RT–qPCR	Viral infectivity, inflammation, high throughput	Gard et al. (2021)
Lung airway	Asthma exacerbation by virus infection	human primary airway epithelial cells, human microvasculature endothelial cells, Immune cells	PDMS 2-Channel Microporous	1 dyn/cm2	60 μL/h	60 μL/h	live human rhinovirus	IFN-λ1, CXCL10 and IL-6	Human neutrophil diapedesis assay, immunostaining, and RT–qPCR	Drug efficacy	Nawroth et al. (2020)
Lung airway	Virus infection (influenza, SARS-CoV-2, MERS-CoV), inflammation	Primary human lung airway epithelial basal stem cells, primary human pulmonary microvascular endothelial cells, primary human alveolar epithelial cells	PDMS 2-Channel Microporous	nil	60 μL/h	60 μL/h	SARS-CoV-2, human 400 coronavirus HCoV-NL63, influenza A/WSN/33 (H1N1), and influenza A/Hong Kong/8/68	IFN-β	RNA-seq, qRT-PCR, gene ontogeny analysis, immunostaining, and western blotting	RNA therapy efficacy	Si et al. (2021b)
BBB	Huntington disease, MCT8 deficiency	iPSC-derived brain microvascular endothelial-like cells, neurons, primary human brain astrocytes, human primary brain vascular pericytes	PDMS 2-Channel Microporous	0.01 dyn/cm2 0.5 dyn/cm2 2.4 dyn/cm2	nil	nil	Plasma/blood	GFAP, a-SMA, and PDGFRb	Permeability assays, viability assay immunostaining, transcriptional and gene ontology analysis	Blood toxicity	Vatine et al. (2019)
BBB	Fungal meningitis	endothelial cells, pericytes, human neural stem cells, fungal	PDMS 3-Channel ECM gel	0–6 dyn/cm−2	nil	nil	C. neoformans	SERPINE1, tissue inhibitor of metalloproteinases-1 (TIMP-1), pentraxin 3 (PTX3), endothelin-1 (ET-1) and thrombospondin-1 (TSP-1)	Immunohistochemistry, qPCR, permeability analysis, RNA seq, cell viability assay, and fungal BBB invasion assay	Fungal invasion of BBB	Kim et al. (2021b)
BBB	Parkinson disease	iPSC-derived dopaminergic neurons, primary human astrocytes, primary human brain pericytes, primary human brain microglia, human brain microvascular endothelial cells	PDMS 2-Channel Microporous	nil	30 μL/h	60 μL/h	αSyn fibril	COL3A1, CENPE, KIF15 and SERPINA1	Mitochondrial membrane potentials assay, western blotting, permeability analysis, cathepsin D activity assay, ELISA and immunostaining	α-Synuclein pathology	Pediaditakis et al. (2021)
Mammary gland	Breast cancer	Human mammary epithelial cells, human dermal microvascular endothelial cells	2-Channel in an ECM gel	∼3.3 Dyn/cm2	nil	∼1.06 μl/s	Basement membrane ECM	IL-6	Permeability assays, conditioned medium assays and immunostaining	Mutation-induced cancer progression, angiogenesis	Kutys et al. (2020)
Heart	Arrhythmia	Primary porcine aortic valvular interstitial cells, valvular endothelial cells	PDMS 2-Channel Microporous membrane	20 dynes/cm2 or 0 dynes/cm2	1.3 μL/min	1.3 μL/min	nil	α-SMA	Immunostaining	cardiovascular cell-cell interactions	Chen et al. (2013)
Heart	Ischemia	Mouse atrial cardiomyocytes	PDMS 1-channel	nil	40 μL/h	nil	hypoxic (1% O2) media	HIF-1∝, α-actinin, and Cx-43	Immunostaining and immunohistochemistry	Hypoxia	Liu et al. (2020)
Eye	Retinopathy	Retinal pigment epithelial cells, hiPSCs-derived retinal organoids (ganglion cells, bipolar cells, horizontal cells, amacrine cells, Müller glia and photoreceptors)	PDMS 2-Channel Nanoporous	nil	20 μl/h	20 μl/h	chloroquine and gentamicin.	PNA lectin, PAX6, MITF, Melanoma gp100, Ezrin and VEGF A	Phagocytosis assay using bovine ROS, VEGF-A secretion assays, live cell endocytosis and phagocytosis assay, immunohistochemistry, qRT-PCR and immunostaining	Drug toxicity	Achberger et al. (2019)
Pancreas	Diabetes mellitus	Human cadaveric islets, stem cell derived β	Thermoplastic 1-Channel	∼6 mPa	1.6 μL min−1	nil	Glucose	Insulin	Immunostaining	Glucose-sensitive insulin secretion	Glieberman et al. (2019)
Kidney glomerulus	Hypertensive nephropathy, mechanosensitivity	glomerular endothelial cells, podocyte	PDMS 2-Channel Nanoporous	0.001, 0.002 and 0.003 dyn/cm2	5 μL/min	5, 10, 15 μL/min	nil	F-actin, insulin, CD-31 and synaptopodin	ELISA, permeability assay, immunohistochemical analyses, immunohistofluorescence analyses and immunostaining	Replication of clinical phenotype	Zhou et al. (2016)
Kidney	Renal hypoxic reperfusion injury	Primary human renal proximal tubular epithelial cells, primary human endothelial cells	Glass 3-Channel Microporous	0.2 dyn/cm2	25 μL/min	45 μL/min	Hypoxic and normoxic cell culture media	KIM-1, HSP70, IL-6 and ET-1	Permeability assays, ELISA, live/dead assay, dichlorodihydrofluorescein diacetate staining	Ischemia, renal hypoxic injury, kidney transplant, graft survival	Chethikkattuveli Salih et al. (2022)
Kidney tubule	Lowe syndrome, Dent II disease	Epithelial cells, proximal tubule cells	Plastic 2-Channel ECM gel	0.06–0.3 dyn/cm2	nil	nil	OCRL protein knockout	SNAI2, TAGLN, COL1A1, COL5A1 and MMP1	Homogenous Time Resolved Fluorescence (HTRF) assay, TGF-β1 trigger and collagen deposition assay, barrier integrity assay, T7E1 assay, immunostaining, RNA Seq and qPCR	Replication of clinical phenotype	Naik et al. (2021)
Kidney tubule	Viral infection	Distal renal tubule epithelial cells	PDMS 2-Channel Nanoporous	nil	11.3 μL/h	nil	Pseudorabies Virus	ZO-1, Na+-K+- and ATPase	Immunofluorescence staining and western blot analysis	Replication of clinical phenotype	Wang et al. (2019)
Kidney tubule	Renal transport, hyperglycaemia	Proximal tubule epithelial cells, glomerular microvascular endothelial cells	3D printed in ECM gel	0.3 dynes/cm2	3 μL/min	3 μL/min	glucose	Ki-67 and SGLT2	Albumin uptake assay, glucose reabsorption assay, permeability assays, immunostaining, direct staining, hyperglycemia assay, VEGF and ELISA	Renal reabsorption, drug efficacy	Lin et al. (2019)
Blood vessel	Progeria, inflammation, mechanosensitivity	Aortic Smooth Muscle cells derived from human induced pluripotent stem cells of HGPS	PDMS 1-Channel	nil	100 µL h−1	100 µL h−1	pathological strain, angiotensin II.	CAV1, SOD1, NADPH, IL6, IL1B, and JUN	Immunohistochemical staining and immunostaining	Drug efficacy	Ribas et al. (2017)
Blood vessel	Breast cancer	Human dermal lymphatic endothelial cells, breast cancer cells	PDMS 3-Channel ECM gel	nil	nil	0.49 to 0.09 μm/s	VEGF-C VEGF-A	CCL21 and CCR7	Cytokine analysis- ELISA, immunofluorescence staining and quantitative mRNA expression analysis	Breast cancer lymph angiogenesis	Cho et al. (2021)
Blood vessel	Colorectal cancer	Human endothelial colony-forming cell-derived endothelial cells, normal human lung fibroblasts, colorectal cancer cells	PDMS 3-Channel ECM gel	nil	nil	nil	nil	COL1A1, COL1A2, COL6A2, ANGPT2, NID2 APLN, CYTL1, DLL4, and IGFBP4	Immunofluorescence, NanoString PanCancer human pathways assay and single cell sequencing	Drug efficacy	Hachey et al. (2021)
Blood vessel	Radiation injury, Shwachman–Diamond syndrome	Mononuclear cells, hematopoietic CD34^+^ cells, bone marrow stromal cell, human umbilical vein endothelial cells	PDMS 2-Channel ECM gel	nil	nil	72 μl/h	Chemotherapeutic Drugs (5-fluorouracil (5-FU)) and ionizing radiation	CD71/CD235, CD41, and CD45	Colony-forming unit assays, flow cytometry analysis, immunofluorescence assay, cell growth assay and Wright–Giemsa staining	Mimic drug toxicity with clinical exposure profiles	Chou et al. (2020)
Blood vessel	Atherosclerosis, vascular stenosis	Primary human umbilical vein endothelial cells, human aortic smooth muscle cell, immune cells	PDMS Multichannel	0.24 Pa	nil	795 μL/min	tumor necrosis factor (TNF)-α	ICAM-1 and vWF	Immunofluorescence staining	Vascular inflammation	Cho and Park (2021)
Blood vessel	Thrombosis	HUVECs	PDMS 2-Channel Microporous	5 dyne/cm2	nil	60 μl/min	anti-CD154 Monoclonal Antibody human whole blood	Thrombin antithrombin complex (TAT), plasminogen activator inhibitor-1 (PAI-1) and SERPINE-2	Platelet activation assays and immunostaining	mAb toxicity	Barrile et al. (2018)
Nerve	Motor neuron injury	iPSC-derived motor neuron progenitors	Plastic 3-Channel ECM gel	nil	nil	nil	glutamate or sodium arsenite vincristine	SMI32, islet-1 (ISL1), choline acetyltransferase (CHAT), ISL1, CHAT, NFH, SLC18A3, VACHT, S100β, MAP2 and axonal marker microtubule-associated protein TAU (MAPT)/TAU	LDH activity assay, qPCR, excitotoxicity assay and immunocytochemistry	Drug toxicity and efficacy	Spijkers et al. (2021)
Gut	Obesity	Primary human adipocytes	PDMS 2-Channel Nanoporous	nil	20–40 μl/h	nil	isoproterenol	lactate dehydrogenase	Live/dead staining, fluorescent double staining, and cytotoxicity analysis	Drug efficacy	Rogal et al. (2020)
Gut	Inflammatory bowel disease	Human colon crypt-derived epithelial cells, colonic human intestinal microvascular endothelial cells	PDMS 2-Channel Microporous	nil	60 mL/h	60 mL/h	interleukin 22	STAT3, caspase 3, interleukin 22, Alpi, Muc2, ChgA, Lgr5, and Bmi1	Permeability assay, immunostaining, Reverse Transcription, and qPCR	Inflammation-associated injury	Apostolou et al. (2021)
Gut	Colorectal cancer	Intestinal epithelial cells, human umbilical vein endothelial cell, cancer-associated fibroblasts, colorectal cancer cells	PDMS 2-Channel Microporous	nil	30 μL h −1	30 μL h −1	Cancer-associated fibroblasts	ZO-1, vimentin and E-cadherin	CAF secretome analysis, tumor cell invasion assay, permeability assay, metabolic analyses RT-qPCR and immunofluorescence	Drug efficacy	Strelez et al. (2021)
Gut	SARS-CoV-2 virus infection	Intestinal epithelial cells Human umbilical endothelial cells human colorectal adenocarcinoma grade II (HT-29) cells immune cell Virus	PDMS 2-Channel Microporous	nil	200 μL/h	50 μL/h	SARS-COV-2 strain 10	NF, IL-6, CXCL1, CXCL10, CXCL11, CCL5, and CSF3	Immunostaining, RNA sequencing and Real-time quantitative PCR	Infection-associated injury, inflammation	Guo et al. (2021)
Gut	Inflammatory bowel disease	Human intestinal crypt-derived epithelial cells Human peripheral blood mononuclear cells	ECM gel 3-Channel	nil	nil	nil	lipopolysaccharide (LPS) interferon-gamma (IFN-γ),	CXCL10, IL-8 and CCL-20	Cytokine assay, viability assay, immunocytochemistry, RNA-seq, Reverse transcription and qRT-PCR	Transcriptome profile	Beaurivage et al. (2020)
Gut	Environmental Enteric Dysfunction	Human intestinal organoid-derived epithelial cells	PDMS 2-Channel Microporous	nil	60 μl/h	nil	niacinamide and tryptophan	neuregulin-4, SMOC2, VNN1, apolipoprotein B, MUC5AC, CLCA1, and CLDN10	Permeability assay, microarray sequencing, and immunofluorescence staining	Clinical phenotype and nutritional dependency	Bein et al. (2022)
Cartilage	Osteoarthritis	Synovial fluid, synovial fibroblast, human primary monocytes articular chondrocytes, human umbilical vein endothelial cells	PDMS 2-Channel 3 ECM gel channels	1–5 dyne cm−2	nil	5, 10, 15, or 30 µl h−1	Monocyte TNF-α	ICAM-1 and VCAM-1	Permeability and chemokine diffusion assay	Monocyte extravasation, drug efficacy	Mondadori et al. (2021)
Liver	Non-alcoholic steatohepatitis	Hepatocyte, liver sinusoidal endothelial cells, hepatic stellate cell, kupffer cell	Plastic 3-Channel ECM gel	nil	nil	nil	LPS and elafibranor oleic acid	Albumin, urea, α-SMA, collagen 1A1 [COL1A1] and TIMP-1	ELISA, immunofluorescence staining, live/dead assay, and neutral lipid staining	Drug efficacy	Freag et al. (2021)
Liver	Steatosis	Human hepatocyte, microvascular cardiac endothelial cells	Plastic 1-Channel	nil	2 μL/min	2 μL/min	Valproate and stavudine	CPT1,COX2, UPC2, and CYP2E1	Live/dead cytotoxicity assay and qRT-PCR	Drug toxicity	Ehrlich et al. (2018)
Liver	Virus (Hepatitis B) infection, inflammation	primary human hepatocyte, kupffer cells	Plastic 1-Channel	nil	1.0 μL/s	nil	hepatitis B virus	IL-6 and TNF-α	CYP450 activity, ELISA, cell viability analysis, cytokine XL and phospho-kinase arrays and immunofluorescence assay	Viral infection-associated injury	Ortega-Prieto et al. (2018)

Drug toxicity, efficacy, and PK-PD modeling: Study of pharmacokinetic and pharmacodynamic (PK/PD) distribution of a drug to reach its target site by satisfying the principle of ADME by utilizing animal models is the biggest challenge faced by scientists these days. With the advancements in microphysiological models, utilizing fluidically interconnected multi-organ human body-on-a-chip platforms, there are possibilities that mimicking of the complex physiological and p0athophysiological biological systems can be done to deeply study the distribution of a drug along with its ADME parameters (Figure 4). Talking of complex physiological systems mimicry, there are a handful of remarkable works regarding multiorgan chips that had been developed in the recent past years. Replicating the absorption, distribution and clearance of a drug has been successful in multi-organ chips, but in other *in vitro* models and single organ chips, the same is not credible. On the other hand, organ chips have always been useful in studying drug efficacy and safety simultaneously. For instance, Hubner et al. administered recurring doses of the anti-EGFR-antibody cetuximab for studying combination of a metastatic tumor environment with a miniaturized healthy organotypic human skin equivalent (Hübner et al., 2018). Similarly, Lee-Montiel integrated a newly established liver-on-a-chip platform with a cardiac-organ on chip platform both of which were created from the same hiPSC line to study drug-drug interaction (Lee-Montiel et al., 2021). Another area where PK/PD models are employed is in microfluidic systems with multi-culture chambers where the culture medium is transported from one compartment to another via a single channel. However, lately separated flow chambers with intervening porous membrane. Herland et al. predicted PK parameters for orally administered nicotine (using gut-on-organ chip, liver-on-organ chip and kidney-on-organ chip) and for intravenously infused cisplatin (using coupled bone marrow-on-organ chip, liver-on-organ chip and kidney-on-organ chip) (Herland et al., 2020).

Computational simulation of drug PK/PD parameters by integrating them to toxicity studies is an interesting and physiologically relevant approach being used these days. Using a multi-organ-organ on chip platform first-pass model made up of interconnected dual-channel human liver-on-a-chip platform, kidney-on-a-chip platform, and intestine-on-a-chip platform as well as an AV reservoir in conjunction with physiologically based computational PK modeling, it has been possible to quantitatively predict human clinical pharmacokinetics parameters (Figure 4). Successful PK parameters translation from *in vitro* to *in vivo* closely resembled clinically observed outcomes (Herland et al., 2020). (Different organs and associated drug toxicity in organ on chip platform have been summarized in Table 2).

**TABLE 2:** Organ on chip platform and drug toxicity.

Organ	Toxicity	Platform/material	Cells	Biomarker	Assays	References
Lung alveolus	Nanoparticle delivery and toxicity	PDMS 1-Channel	Adenocarcinoma human alveolar basal epithelial cells, mouse fibroblast cell	Lactate dehydrogenase, ROS, Bax and Bcl-2	LDH assay, RT-qPCR, Rhodamine Phalloidin staining, JC-1 staining, acridine orange assay, DAPI staining, PI staining, flow cytometry, Annexin V/PI flow cytometric assay, MTT assay, neutral red uptake assay DCFH-DA assay and Griess test	Arathi et al. (2022)
Lung alveolus	Breathing-dependent nanoparticulate toxicity	PDMS 2-Channel Microporous	Human alveolar epithelial cells, human pulmonary microvascular endothelial cells, immune cells	VE Cadherin, occludin, ICAM-1 and ROS	Permeability assay, immunohistochemistry, Immunofluorescent staining and caspase assay	Huh et al. (2010)
Heart	Heart contractility, cardiotoxicity	PDMS + plastic 3D printed	HUVECs, hiPSC-cardiomyocytes	F-actin, CD31, sarcomeric α-actinin, connexin-43 and vWF	ELISA, Immunofluorescent staining, and live/dead staining	Zhang et al. (2016)
Heart	cardiotoxicity	Plastic 1-Channel	Human induced pluripotent stem cell-derived cardiomyocytes	a-actinin	Immunofluorescent staining	Kujala et al. (2016)
Kidney glomerulus	Personalized drug testing	PDMS 2-Channel Microporous	Human iPSc derived vascular endothelial cell, human iPSc derived pdocytes, intermediate mesoderm (IM) cells	Albumin, VEGF 165, podocin, nephrin, collagen IV, VE-Cadherin, inulin and PECAM-1	VEGF-A ELISA and Immunofluorescent staining	Roye et al. (2021)
Kidney glomerulus	Filtration barrier, autoimmune toxicity	Plastic 3-Channel ECM gel	Podocytes, human lines of fibroblasts (hFIB), human lung endothelial cells (HuLECs), human glomerular endothelial cells	CD31, WGA, COL4A4, COL4A12, LAMA5A, beta actin, nephrin, F-actin, Albumin, NPHS1	Albumin assay, flow cytometry, Immunofluorescent staining, western blot analysis and inulin permeability assay	Petrosyan et al. (2019)
Kidney glomerulus	Filtration barrier	PDMS 2-Channel Microporous	human induced pluripotent stem (hiPS) cells, podocytes, human microvascular endothelia cells	Oct4, WT1, POU5F1, Pax2, WT1, NPHS1, NPHS2,OSR1, podocin, nephrin, albumin and inulin	EdU-incorporation assay, Immunofluorescent staining, cell-viability assay, western blot analysis, albumin-uptake assay and inulin-and-albumin-filtration assay	Musah et al. (2017)
Kidney glomerulus	Nephrotoxicity	Plastic 3-Channel ECM gel	proximal tubule epithelial cell	Albumin, ZO-1, Ezrin, VE-cadherin, P-glycoprotein, LDH, N-acetyl-β-d-glucosaminidase, mir-192, mir-34a, mir-21 and mir-29a	LDH activity assay, immunohistochemistry, Caspase-3/7 assay, WST-8 assay and Immunofluorescent staining	Vormann et al. (2022)
Kidney tubule	Renal transport, nephrotoxicity	3D printed in ECM gel	proximal tubule epithelial cells	Na+/K+ATPase, laminin, collagen IV, actin, tubulin, K Cadherin, albumin, megalin, lotus tetragonolobus lectin and Aquaporin 1 (AQP1)	Cytokine analysis, gene expression analysis, permeability assay, immunofluorescent staining and albumin uptake.	Homan et al. (2016)
Kidney tubule	Renal transport, nephrotoxicity	PDMS 2-Channel Microporous	Primary human kidney proximal tubular epithelial cells	LDH, Na/K-ATPase and AQP1, sodium glucose cotransporter 2 (SGLT2), a-tubulin, alkaline phosphatase (ALP), albumin	Albumin-uptake assay, western blot, glucose assay and ALP assay	Jang et al. (2013)
Liver	human drug toxicities in DILI	PDMS 2-Channel Microporous	Primary human hepatocytes, human liver sinusoidal endothelial cells, human Kupffer cells, human Stellate cells	Albumin, urea, actin, MRP2, desmin, ATPB, IL-6 and TNFa	Albumin assay, alanine transaminase (ALT), cytotoxicity assay, live staining, immunofluorescent staining, next-generation sequencing (NGS) and cytokine analysis	Ewart et al. (2022)
Liver	Hepatotoxicity	Plastic 2-Channel ECM gel	iPSC derived hepatocytes, human microvascular endothelial cell, Immune cells	Albumin, urea, LDH, alpha-fetoprotein (AFP), CYP3A4, F-actin, and CD68	Albumin assay, Luciferin- IPA assay, cell viability assay and LDH assay	Bircsak et al. (2021)
Liver	Human and cross-species drug toxicities in DILI	PDMS 2-Channel Microporous	Primary hepatocytes, liver sinusoidal endothelial cells, Kupffer, stellate cells	α-sma, ALT, AST, albumin, miR122, lipids, IP-10, IL-6, MCP-1, ATP, BSEP, CLF, CDFDA, ∝-GST CD68, and keratin 18	Cytochrome P450 enzyme activity, albumin assay, AST, ALT, ATP GSH, GLDH, and ELISA	Jang et al. (2019)
Liver	Drug- and toxin-induced liver injury	Plastic 1-Channel	human hepatocytes	Albumin, Urea, LDH, WST-1, Total Protein, GSH,SOD1	Albumin assay, CDFDA staining of bile canaliculi, transcriptional analysis, radioactive cytotoxicity assay, glutathione Assay, and ELISA	Rowe et al. (2018)
Liver	Metabolism-dependent toxicity	PDMS 2-Channel Microporous	Rat and human primary hepatocytes	nil	Cell viability staining	Lee et al. (2007)
Liver	Drug induced liver injury	PDMS 2-Channel Microporous	induced pluripotent stem cells (iPSC), primary human hepatocytes	ALT, AST, albumin, cytochrome P450	Cytochrome P450 enzyme activity, albumin assay, AST, ALT, scRNA sequencing, cytotoxicity assays and immunostaining	Zhang et al. (2023)
Teeth	Dental material toxicities	PDMS 3-Channel Dentin fragment	Stem cells from apical papilla, dentin	Dentin and actin	Live cell and cytotoxicity staining	França et al. (2020)
Skin	Skin irritation due to drug and chemical toxicity	Plastic 2-Chamber Nanoporous	normal human keratinocytes	IL-6, TNF-α, ZO-1, CK 10 (keratin-10), CK 19, CK 14 (keratin-14), involucrin, loricrin, and filaggrin	Histological and immunofluorescent analysis, permeability assay and ELISA	Zhang et al. (2021b)
Immune system	Tumor-targeted T cell bispecific Ab toxicity	PDMS 2-Channel Microporous	Human alveolar epithelial cells, human microvascular lung endothelial cells, peripheral blood mononuclear cells (PBMC), intestinal tumor cells	E- cadherin, granzyme B, CEA, IFNγ, IL-2, IL-6, IL-8, IL-10, IL-13, IL1RA, TNFα, MIP-1β, G-CSF, and GM-CSF	Cytokine analysis, immunohistochemistry, flow cytometry and live staining	Kerns et al. (2021)

### Toxicity and organ on chip

Organ-on-chip platform has emerged as a promising tool to assess the toxicity of drugs and chemicals. Organ-on-chip platforms have the potential to offer several advantages over traditional *in vitro* and *in vivo* models, including higher throughput, increased reproducibility, and greater predictive power. This approach has been applied to evaluate toxicity in various organs, including the liver, lung, kidney, and heart. In this paragraph, we will discuss the application of organ-on-chip platforms for toxicity evaluations (Cong et al., 2020).

Lung toxicity: Arathi et al. conducted a microfluidic study to investigate the toxicity of Cys-ZnO nanoparticles, and discovered that their toxicity was dependent on parameters such as perfusion, exposure time, and local concentration. The study utilized a single-channeled microfluidic device made of PDMS, along with Adenocarcinoma human alveolar basal epithelial cells (A549 cells) and mouse fibroblast cells. The results showed that increasing concentrations (up to 160 μg/mL) of Cys-ZnO nanoparticles induced apoptosis, as evidenced by LDH leakage, depolarization of mitochondrial membrane, blebbing, actin filament condensation, nuclear condensation, ROS production, lysosomal damage, upregulation of Bax gene expression, and downregulation of Bcl-2 gene expression. The study provides insight into the toxic effects of Cys-ZnO nanoparticles, and highlights the utility of microfluidic devices in examining nanoparticle toxicity (Arathi et al., 2022). Huh *et al.* developed a lung-on-a-chip platform to study the pulmonary response to nanoparticles delivered to the epithelial compartment in the form of an aerosol. The platform consists of a two-channeled PDMS microfluidic device separated by a microporous membrane to model the lung alveolus. The authors investigated the effects of cyclic mechanical strain on the lung inflammatory and toxicity reactions to silica nanoparticles using HPAEpics, HMPECs, and immune cells. The study revealed that the responses to silica nanoparticles were amplified by cyclic mechanical strain, as evidenced by elevated levels of the biomarkers ROS and ICAM-1. This platform represents an innovative tool for the study of nanotoxicology in the lung and could provide insights into the mechanisms of pulmonary diseases induced by exposure to nanoparticles (Huh et al., 2010).

Cardiac toxicity: Zhang et al. introduced a new hybrid approach for generating endothelialized myocardial tissues using 3D bioprinting. The strategy involved directly encapsulating endothelial cells within the bioprinted microfibrous lattices to encourage migration towards the periphery of the microfibers for the formation of a confluent endothelium. The team combined this with a custom-made microfluidic perfusion bioreactor to evaluate the cardiovascular effects of pharmaceutical compounds. The study employed a 3D printed microfluidic device made of plastic and PDMS, containing cultured HUVECs and hiPSC-cardiomyocytes. The team exposed the endothelialized myocardium on a chip platform to doxorubicin, a conventional anti-cancer drug, and observed reduced heart beat rates and lower levels of the biomarker vWF secreted by the ECs (Zhang et al., 2016; Park et al., 2019). Kujala et al. demonstrated the potential of soft micro molded gelatin with topographical cues to direct hiPSC-CMs towards the formation of laminar cardiac tissues resembling the native architecture of the heart. The researchers utilized a single channeled microfluidic device with a collagen-based extracellular matrix and physiologically relevant substrate stiffness to maintain electrophysiological activity while allowing for drug studies. Baseline cardiac field potentials were recorded and compared with those obtained after pharmacological intervention with isoproterenol, confirming the platform’s validity. Furthermore, the system’s capability of predicting human laminar cardiac tissue response to a cardiotoxic pro-drug (terfenadine) and its non-cardiotoxic metabolite (fexofenadine) was evaluated. The study employed human induced pluripotent stem cell-derived cardiomyocytes, making it a promising tool for drug testing and cardiac disease modeling (Kujala et al., 2016).

Renal Toxicity: Roye and colleagues developed a dual-channel PDMS-based glomerulus-on-a-chip platform that closely mimics the key structural and functional features of the kidney glomerular filtration barrier using human induced pluripotent stem cell-derived endothelial cells, podocytes, and intermediate mesoderm cells. In their study, the platform was perfused with the chemotherapeutic drug Adriamycin for up to 48 h to investigate its effects on the structural integrity of the endothelium and podocyte tissue layers, leading to albuminuria, a hallmark of glomerulopathy. The authors also investigated biomarkers such as Albumin, VE-Cadherin, and PECAM-1 in the two-channeled PDMS microporous microfluidic device, confirming the platform’s suitability for disease modeling and drug screening in kidney-related diseases (Roye et al., 2021). Petrosyan et al. developed a glomerulus-on-a-chip platform to mimic the structure and function of the kidney glomerulus using a multi-channeled extracellular matrix (ECM) containing plastic microfluidic device. Human glomerular endothelial cells (hGEC), human fibroblasts (hFIB), human lung endothelial cells (HuLECs), and human podocytes were seeded on the platform to create a layer of ECM, which included laminin and collagen IV trimer, the two primary components of the glomerular basement membrane (GBM) *in vivo*. The study showed that the cells maintained their phenotypic characteristics and interacted appropriately with each other for an extended period. The platform was evaluated using serum samples collected from patients with various glomerular diseases, including membranous nephropathy (MN), to assess drug responses. The study also demonstrated the detection of albumin leakage, a biomarker of glomerular disease, in the glomerulus-on-a-chip platform (Petrosyan et al., 2019). Musah et al. developed a glomerulus-on-a-chip platform by co-culturing hiPSC-derived podocytes with kidney glomerular endothelial cells (GECs) in a double-channeled PDMS-based microporous device. Their platform could replicate the molecular filtration capacity of the glomerular capillary wall and tissue-tissue interactions. Moreover, their glomerulus model could reproduce proteinuria and podocyte injury induced by the drug Adriamycin *in vitro*. The study evaluated the biomarkers albumin and inulin (Musah et al., 2017). In their study, Vormann *et al.* aimed to develop a high throughput, multi-channeled plastic-based organ on chip platform called Nephroscreen for the pharmaceutical industry to detect drug-induced nephrotoxicity. The authors evaluated four different drugs, namely cisplatin, tenofovir, tobramycin, and cyclosporin A, which are known to cause nephrotoxicity. They analyzed various characteristics during the study, including cell survival, barrier integrity, release of lactate dehydrogenase (LDH) and N-acetyl-b-D-glucosaminidase (NAG), specific miRNA release, and expression of toxicity markers. The study also identified drug-transporter interactions for P-gp and MRP2/4. The cells used in the development of Nephroscreen were proximal tubule epithelial cells. The study shows that the Nephroscreen platform could be a useful tool for the pharmaceutical industry to predict drug-induced nephrotoxicity (Vormann et al., 2021). Homan *et al.* have reported on a promising bioprinting technique for fabricating 3D human renal proximal tubules (PCT) *in vitro* that are embedded within a supportive extracellular matrix (ECM) and preserved in a perfusable organ-on-a-chip platform for more than 2 months. The team constructed the PCT architecture, which is enclosed by proximal tubule epithelial cells (PTEc) and consistently perfused through the open lumen. Compared to 2D cell culture with and without perfusion, their 3D PCT-on-a-chip platform exhibited significantly enhanced epithelial morphology and functional characteristics. They also administered the nephrotoxin Cyclosporine A in a dose-dependent manner and observed an epithelial barrier breakdown. During the study, biomarkers such as Na+/K+ATPase, lotus tetragonolobus lectin, and Aquaporin 1 (AQP1) were evaluated (Homan et al., 2016). Jang *et al.* conducted a study to investigate toxicity in a proximal tubule-on-a-chip platform using renal proximal tubule epithelial cells (RPTECs) seeded on the apical part of an extracellular matrix (ECM)-coated, porous membrane that divides the device’s main channel into two neighboring channels, resulting in a “luminal” top channel and a bottom “interstitial” area. Compared to conventional Transwell culture techniques, exposure of the epithelial monolayer to an apical fluid shear stress that resembles that seen in the *in vivo* kidney tubules resulted in improved epithelial cell polarization and primary cilia development. In addition, in their study, the cells showed noticeably higher glucose uptake, albumin transport, and activity of brush border alkaline phosphatase. They found that measurements of cisplatin toxicity and Pgp efflux transporter activity on the organ-on-chip platform more accurately reflect the results seen *in vivo* than data obtained with cells maintained under traditional culture conditions (Jang et al., 2013).

Liver toxicity: In the pursuit of developing reliable platforms for predicting drug-induced liver injury (DILI), Ewart *et al.* evaluated commercially available human Liver-Chips. The team tested 27 different drugs on 870 Liver-Chips and compared the findings to previous results from animal models and spheroids of primary human hepatocytes. The aim was to investigate the suitability of Liver-Chips for preclinical research, particularly in support of toxicity-related decisions. The authors also discussed how pharmaceutical sector screening programs could potentially use this platform. To achieve this, a PDMS device with two channels and a microporous membrane in between was used to maintain a flow rate of 30 μL/h in the apical and basal chamber of the chip. The biomarkers analyzed in this study were albumin, urea, IL-6, and TNFα. The team’s research has implications for the development of effective DILI screening strategies and highlights the potential of the Liver-Chip as a predictive *in vitro* tool for evaluating liver toxicity (Ewart et al., 2021). Bircsak et al. developed a liver organ-on-chip platform for high throughput screening of hepatotoxicity. They used iPSC iCell 2.0 hepatocytes lined in a 96-well Mimetas OrganoPlate 2-lane to form a vascular channel. The researchers performed dose-response assessments with different drugs and validated the results through albumin assay, which was found to be the most sensitive hit and helped to calculate TC50 values. The authors recommend that 3-dimensional complex liver models for hepatotoxicity testing should include not only hepatocytes but also innate immune cells. This approach can provide valuable information for predicting toxicity and drug safety, as well as for developing new drugs. This platform could be useful in the pharmaceutical industry for preclinical drug testing and could help reduce the use of animal models in drug development. The biomarker evaluated in the study was albumin (Asif et al., 2021; Bircsak et al., 2021). In their study, Jang *et al.* developed a PDMS-based organ-on-chip platform for investigating species-specific drug-induced liver injury, fibrosis, cholestasis, and steatosis. This platform was lined with rat tissue, dog tissue, and species-specific human primary hepatocytes, stellate cells on the apical part, and liver sinusoidal endothelial cell and Kupffer cell on the basal part, at a physiologically relevant fluidic flow rate of 10 μl/h. The study investigated the effects of different drugs and different concentrations, including bosentan, APAP, JnJ-1, JNJ-2, JNJ-3, TAK-875 MTX, and FIAU. The researchers validated different biomarkers such as α-sma, ALT, AST, albumin, miR122, lipids (nile red), IP-10, IL-6, MCP-1, ATP, BSEP, CLF, CDFDA, CD68, and keratin 18. These biomarkers were used to evaluate liver function and toxicity under different experimental conditions (Jang et al., 2019; Kim et al., 2021c; Farooqi et al., 2021). Rowe *et al.* conducted a study highlighting the potential efficacy of perfused human liver microtissues for drug development and mechanistic toxicology using an organ-on-chip platform. The platform was lined with human primary hepatocytes and was exposed to compounds that form reactive metabolites and disrupt mitochondria. The study employed the Plastic 1 channel with human hepatocyte cell lines and used xenobiotics and hepatotoxins as inducers with a 2 μL/min flow rate of endothelial and epithelial cells. The authors identified several biomarkers, including Albumin, Urea, LDH, WST-1, Total Protein, SOD1, and GSH. The study evaluated the model’s phenotypic stability, metabolic function’s applicability to the human liver, and reactions to recognized hepatotoxins that generate reactive metabolites or damage mitochondria. The results showed that the platform is metabolically functional and has the potential to be an effective tool for drug development and mechanistic toxicology. The study further emphasized the importance of identifying biomarkers for the assessment of the model’s efficacy in drug development and mechanistic toxicology (Rowe et al., 2018). Lee *et al.* proposed an innovative microfluidic platform for primary hepatocyte culture that mimics the mass transport properties of the functional liver sinusoid. The system includes an endothelial-like barrier that enables extensive cell-cell contact, defined tissue and fluid transport regions, and continuous nutrient exchange, facilitating improved functionality of the cultured cells. The study utilized rat and human primary hepatocytes seeded on a PDMS-2 channel microporous membrane. Remarkably, the cells were able to be maintained for 7 days without a reduction in viability. This platform offers a valuable tool for studying liver physiology, drug metabolism, and toxicity, and may ultimately lead to the development of more accurate *in vitro* models for drug development and mechanistic toxicology (Lee et al., 2007). Charles J. *et al.* demonstrated that liver organoids (HLOs) have the potential to serve as a viable *in vitro* model for drug-induced liver injury (DILI) risk prediction. HLOs are scalable, consistent and amenable to high-throughput screening, making them a suitable model for large-scale DILI risk assessment. Moreover, organoids on chip increase albumin and CYP expression, and respond to hepatotoxic drugs. The authors used phenotypic clustering to identify similarities in drug mechanisms of hepatic injury, which allowed for the prediction of synergistic toxicities. They found that liver chips were able to model the hepatotoxicity of tenofovir-inarigivir combination therapy, which was not detected in previous studies until higher concentrations were reached. Additionally, transcriptomics with morphological profiling was used to predict other synergistic toxicities. Overall, the study highlights the potential of liver organoids as a promising model for DILI risk prediction and emphasizes the need for further optimization and expansion to encompass patient genetic diversity (Ahmed et al., 2022f; Zhang et al., 2023).

Teeth, skin, immune system: Miranda Franca et al. developed a tooth-on-a-chip platform using PDMS-based Dentin fragments and apical papilla stem cells. This platform mimics the physiologically relevant interaction between human teeth and biomaterials. The authors found that this model offers a direct visualization of the complexity of the pulp–dentin–biomaterials interface, and enables real-time monitoring of the pulp cells’ response to dental materials. The platform used in this study was lined with apical papilla stem cells on one side and PDMS-based Dentin fragments on the other. The authors evaluated the direct interaction between the stem cells and the Dentin fragments to determine how they would respond to different biomaterials. They found that the model provides a more accurate representation of the way human teeth interact with biomaterials than traditional *in vitro* models, which lack the complex structure and cellular diversity of the pulp–dentin interface. Furthermore, this model allows real-time monitoring of the interaction between the dental materials and the stem cells, providing valuable information about the biocompatibility and safety of these materials. The authors suggest that this platform has the potential to be used in the development and testing of new dental materials and treatments, as well as in personalized dentistry, where patient-specific dental models can be created for testing different biomaterials (França et al., 2020). Zhang et al. introduced a high-fidelity epidermis organ on chip platform for scalable *in vitro* irritation evaluation, which can support clinically and pathophysiologically relevant 3D complex *in vitro* epidermis organ on chip platform models. The platform utilized normal human keratinocytes on a Plastic 2-Chamber nanoporous platform, and the biomarkers identified were CK 10 (keratin-10), CK 14 (keratin-14), involucrin, loricrin, IL-6, TNF-α, and filaggrin. The authors claim that a dynamic epidermis culture model could enrich the barrier function. This model allows direct visualization of the interactions between the biomaterial and the skin, enabling real-time monitoring of the skin’s response. In addition, this platform can be used for scalable testing, making it an effective tool for *in vitro* irritation evaluation (Zhang et al., 2021b). Kerns et al. developed a human immunocompetent organ on chip platform to evaluate the safety profile of T-cell bispecific antibodies (TCBs) targeting tumor antigens. The study utilized HeLa, MKN45, and HEK293T cell lines on a PDMS 2-chamber microporous platform. The authors identified CD69^+^, killer CD8^+^, IFN-γ, and IL-6 as the biomarkers for this model. They found that the lung and intestine organ on chip platform model could effectively predict and replicate target-dependent TCB safety liabilities. The model demonstrated a sensitive response to key determinants such as target expression and antibody affinity. The authors suggest that including resident immune cells and lymphoid structures, as well as techniques that mimic T-cell trafficking and tissue infiltration, can enhance the predictive power of the platform. This study highlights the importance of incorporating immunocompetent cells and relevant biological structures in organ on chip platforms to improve their utility in drug development and toxicity testing (Kerns et al., 2021).

### Personalized medicine and organ on chip

Around a decade ago, the concept of personalized medicine emerged, providing evidence that drug responses vary with different individuals based on their health, epigenetic factors, age, ongoing therapy, nutrition, and so on (Vogenberg et al., 2010). The emerging field of stem cell and organ on a chip technology permits to model disease and recovery for personalized treatment. The current innovative trend in medical field innovation includes organ on chip and iPSC which focuses on human cell types, organ types and whole human organs integrated with engineering to model physiologically relevant human organs. Furthermore, single organ chip, multi-organ on a chip and human on a chip enables the acceleration of the patient specific personalized medicine and enhance the understanding of the genetic variation, biological factors, and physiology of the disease of the patient. All these parameters are combined to identify potential therapeutic targets. The example of the personalized medicine on a chip is shortly described here, the patients’ whole blood and tissue samples are collected and analyzed for disease severity using mRNA analysis, RNA sequencing and to create the genetic blueprint with the help of organ on chip and determine disease severity (low, moderate, and severe), therapeutic target and treatment. This whole procedure can be performed on the organ on chip.

By combining organ-on-chip technology with personalized medicine, it is possible to identify new therapeutic targets and develop customized treatments that can be tailored to each patient’s specific needs. For instance, patient-specific organs on a chip are being developed to identify drug toxicity and to predict drug efficacy. In a recent study, liver on a chip was developed to evaluate the toxicity of acetaminophen, which is commonly used for pain relief. The study revealed that the toxicity of acetaminophen was dependent on the age and sex of the donors of the liver cells. Such findings could be used to predict the toxicity of acetaminophen in a particular patient and suggest alternative drugs (emulate toxicity paper). Another example of personalized medicine on a chip is the use of a gut on a chip to test the efficacy of cancer drugs in patients with gastrointestinal tumors. The gut on a chip models the tumor microenvironment, enabling researchers to evaluate the effect of drugs on the tumor cells and predict the patient’s response to therapy. In a similar study, a lung on a chip was used to model the effect of cystic fibrosis on the lung cells and to develop personalized treatments for the disease (Liu et al., 2021; Liu et al., 2022b).

Organ on chip technology has also shown great promise in the field of regenerative medicine. For example, in a recent study, researchers used a bone on a chip to develop personalized treatments for bone fractures. The bone on a chip was used to test various drugs and combinations of drugs to identify the most effective treatment for bone regeneration (Kengelbach-Weigand et al., 2021; Mansoorifar et al., 2021; Yun et al., 2021). Similarly, researchers are using heart on a chip technology to develop personalized treatments for heart disease. The heart on a chip can mimic the biomechanical properties of the heart, enabling researchers to test the efficacy of drugs and identify new therapeutic targets for heart disease (Zhang et al., 2015)^,^ (Zhao et al., 2020).

Moreover, the gut-on-a-chip technology has been developed to study the interaction between the gut microbiota and the intestinal epithelium, allowing for personalized treatment of gastrointestinal diseases such as inflammatory bowel disease (IBD) and irritable bowel syndrome (IBS). This technology has also been used to study the effects of dietary factors on gut health, providing insights into the mechanisms underlying gut diseases (Morelli et al., 2023).

**FIGURE 5:**
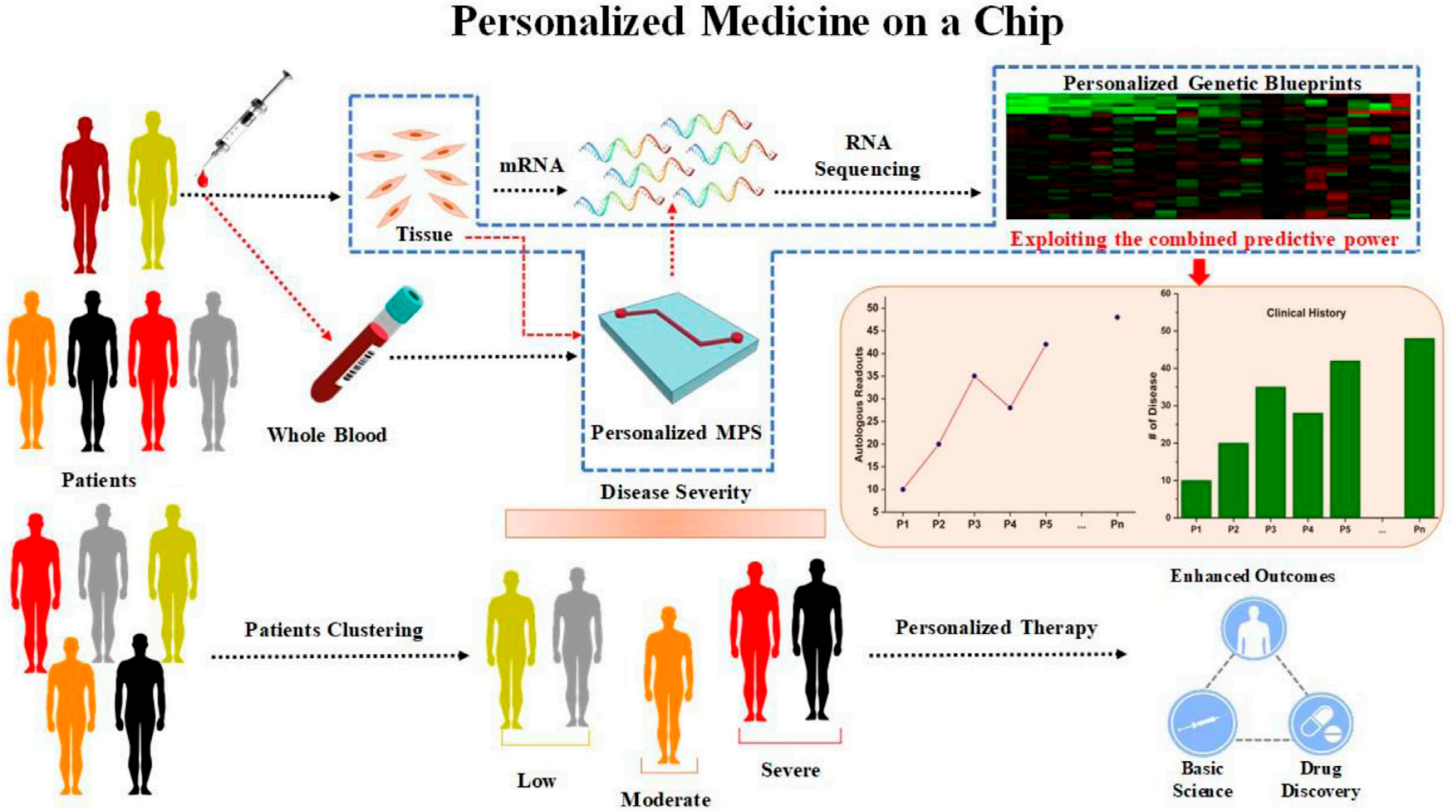
schematic of Personalized Medicine-on-organ on chip platform: Patient-derived cells, such as iPSCs or primary cells, can be used to create personalized-organ on chip platform. RNA sequencing using tissues or cells will reveal the personalized genetic blueprints of each individual patient, promoting genetic disease modeling and toxicity testing. Drug responses or disease severity among individuals in a subpopulation can be modeled using a single organ or an interconnected multiorgan-organ on chip platform lined with cells from individual patients, and the patient population can be grouped based on the personalized results obtained from personalized MPS modeling. This will aid in the drug discovery process.

In conclusion, personalized medicine and organ on chip technology have revolutionized the field of medicine, enabling researchers to model the pathophysiology of human organs and predict patient-specific drug responses. These technologies have great potential in accelerating the development of personalized therapies and in reducing the use of animal models in drug discovery. As organ on chip technology continues to advance, it is expected to become an indispensable tool in the field of medicine. (Personalized Medicine on-organ on chip is summarized in Figure 5).

## Conclusion and future prospects

The fact that human mimicking diseases and related conditions can be developed to study various complex diseases and accompanying abnormalities in varieties of human organ on chip platform models is a well-known point. The benefits of replacing animal models with organ chips are a solid reason why researchers should switch along with the economic importance and clinical relevance of the physiological and pathophysiological models. In addition, since animal physiology is complicated and maintenance of animals for drug testing is a tedious job, drug testing studies can be a failure sometimes. However, there are still some limitations and goals to be achieved in order to make organ chips more popular for research in the field of drug discovery and developing complex disease models.

In this review, we have summarized the parameters used in each relevant study that includes sheer stress, flow rate, cell type, platform, and platform material in an organized and elaborative way. We have also included drug toxicity and Pharmacokinetic/Pharmacodynamic (PK/PD) studies that can test the safety, efficacy and ADME parameters of various drugs targeting a wholesome of both complex and widely studied disease. While pointing out the research gaps and future directions to where organ chips lead us, there are a few to be discussed. For example, limitations of current frequently used platforms like PDMS have an absorption rate of 50%–60%. The introduction of a new, more efficient material to be used as platform is in utter need for improving the system model’s accurate functionality. There are several complex and rare diseases yet to be established on microphysiological systems which can pave a way towards a generous and innovative contribution to the drug discovery sector of microphysiological and tissue engineering. There is an urgent need for standardized platform for modelling or mimicking a specific disease, i.e., for example, a standard unit of platform of liver injury model for liver fibrosis which can be employed by scientists and pharmacological experts all around the world. Real-time monitoring of the cell systems is presently being followed in order to monitor the cells, better changes can be observed if replaced with online monitoring which has been made easier with easier, faster and convenient usage of internet even not while being present at the location. Making ADME properties evaluation a mandatory criterion for every drug testing and disease mirophysiological model. Integration of omics data such as genomics, transcriptomics, or proteomics as an extra validation of the biomarkers identified can be a method of adding more molecular level validation to the results and confirming the biomarker identified as valid.

## References

[B1] Achberger K. Probst C. Haderspeck J. Bolz S. Rogal J. Chuchuy J. (2019). Merging organoid and organ-on-a-chip technology to generate complex multi-layer tissue models in a human retina-on-a-chip platform. eLife 8, e46188. 10.7554/eLife.46188 31451149 PMC6777939

[B2] Ahmed F. Ho S. G. Samantasinghar A. Memon F. H. Rahim C. S. A. Soomro A. M. (2022f). Drug repurposing in psoriasis, performed by reversal of disease-associated gene expression profiles. Comput. Struct. Biotechnol. J. 20, 6097–6107. 10.1016/j.csbj.2022.10.046 36420161 PMC9668643

[B3] Ahmed F. Kang I. S. Kim K. H. Asif A. Rahim C. S. A. Samantasinghar A. (2023). Drug repurposing for viral cancers: A paradigm of machine learning, deep learning, and virtual screening-based approaches. J. Med. Virology 95 (4), e28693. 10.1002/jmv.28693 36946499

[B4] Ahmed F. Lee J. W. Samantasinghar A. Kim Y. S. Kim K. H. Kang I. S. (2022e). SperoPredictor: An integrated machine learning and molecular docking-based drug repurposing framework with use case of COVID-19. Front. Public Health 10, 902123. 10.3389/fpubh.2022.902123 35784208 PMC9244710

[B5] Ahmed F. Soomro A. M. Ashraf H. Rahim A. Asif A. Jawed B. (2022a). Robust ultrasensitive stretchable sensor for wearable and high-end robotics applications. J. Mater. Sci. Mater. Electron. 33 (35), 26447–26463. 10.1007/s10854-022-09324-0

[B6] Ahmed F. Soomro A. M. Chethikkattuveli Salih A. R. Samantasinghar A. Asif A. Kang I. S. (2022d). A comprehensive review of artificial intelligence and network based approaches to drug repurposing in Covid-19. Biomed. Pharmacother. 153, 113350. 10.1016/j.biopha.2022.113350 35777222 PMC9236981

[B7] Ahmed F. Waqas M. Jawed B. Soomro A. M. Kumar S. Hina A. (2022b). Decade of bio-inspired soft robots: A review. Smart Mater. Struct. 31 (7), 073002. 10.1088/1361-665x/ac6e15

[B8] Ahmed F. Waqas M. Shaikh B. Khan U. Soomro A. M. Kumar S. (2022c). Multi-material bio-inspired soft Octopus robot for underwater synchronous swimming. J. Bionic Eng. 19 (5), 1229–1241. 10.1007/s42235-022-00208-x

[B9] Ahmed T. (2022). Organ-on-a-chip microengineering for bio-mimicking disease models and revolutionizing drug discovery. Biosens. Bioelectron. X 11, 100194. 10.1016/j.biosx.2022.100194

[B10] Albanese A. Lam A. K. Sykes E. A. Rocheleau J. V. Chan W. C. W. (2013). Tumour-on-a-chip provides an optical window into nanoparticle tissue transport. Nat. Commun. 4 (1), 2718. 10.1038/ncomms3718 24177351 PMC3947376

[B193] Amin H. H. Meghani N. M. Oh K. T. Choi H. Lee B.-J. (2017). A conjugation of stearic acid to apotransferrin, fattigation-platform, as a core to form self-assembled nanoparticles: Encapsulation of a hydrophobic paclitaxel and receptor-driven cancer targeting. J. Drug. Deliv. Sci. Technol. 41, 222–230. 10.1016/j.jddst.2017.07.013

[B11] Apostolou A. Panchakshari R. A. Banerjee A. Manatakis D. V. Paraskevopoulou M. D. Luc R. (2021). A novel microphysiological colon platform to decipher mechanisms driving human intestinal permeability. Cell Mol. Gastroenterol. Hepatol. 12 (5), 1719–1741. 10.1016/j.jcmgh.2021.07.004 34284165 PMC8551844

[B12] Arathi A. Joseph X. Akhil V. Mohanan P. V. (2022). L-Cysteine capped zinc oxide nanoparticles induced cellular response on adenocarcinomic human alveolar basal epithelial cells using a conventional and organ-on-a-chip approach. Colloids Surfaces B Biointerfaces 211, 112300. 10.1016/j.colsurfb.2021.112300 34974288

[B13] Asif A. Park S. H. Manzoor Soomro A. Khalid M. A. U. Salih A. R. C. Kang B. (2021). Microphysiological system with continuous analysis of albumin for hepatotoxicity modeling and drug screening. J. Industrial Eng. Chem. 98, 318–326. 10.1016/j.jiec.2021.03.035

[B14] Balijepalli A. Sivaramakrishan V. (2017). Organs-on-chips: Research and commercial perspectives. Drug Discov. today 22 (2), 397–403. 10.1016/j.drudis.2016.11.009 27866008

[B15] Barrile R. van der Meer A. D. Park H. Fraser J. P. Simic D. Teng F. (2018). Organ-on-Chip recapitulates thrombosis induced by an anti-cd154 monoclonal antibody: Translational potential of advanced microengineered systems. Clin. Pharmacol. Ther. 104 (6), 1240–1248. 10.1002/cpt.1054 29484632

[B16] Bauer S. Wennberg Huldt C. Kanebratt K. P. Durieux I. Gunne D. Andersson S. (2017). Functional coupling of human pancreatic islets and liver spheroids on-a-chip: Towards a novel human *ex vivo* type 2 diabetes model. Sci. Rep. 7 (1), 14620. 10.1038/s41598-017-14815-w 29097671 PMC5668271

[B17] Beaurivage C. Kanapeckaite A. Loomans C. Erdmann K. S. Stallen J. Janssen R. A. J. (2020). Development of a human primary gut-on-a-chip to model inflammatory processes. Sci. Rep. 10 (1), 21475. 10.1038/s41598-020-78359-2 33293676 PMC7722760

[B18] Bein A. Fadel C. W. Swenor B. Cao W. Powers R. K. Camacho D. M. (2022). Nutritional deficiency in an intestine-on-a-chip recapitulates injury hallmarks associated with environmental enteric dysfunction. Nat. Biomed. Eng. 6 (11), 1236–1247. 10.1038/s41551-022-00899-x 35739419 PMC9652151

[B19] Bein A. Fadel C. W. Swenor B. Cao W. Powers R. K. Camacho D. M. (2021). Nutritional deficiency recapitulates intestinal injury associated with environmental enteric dysfunction in patient-derived Organ Chips. medRxiv. 10.1101/2021.10.11.21264722 PMC9652151 35739419

[B20] Benam K. H. Novak R. Nawroth J. Hirano-Kobayashi M. Ferrante T. C. Choe Y. (2016b). Matched-comparative modeling of normal and diseased human airway responses using a microengineered breathing lung chip. Cell Syst. 3 (5), 456–466.e4. 10.1016/j.cels.2016.10.003 27894999

[B21] Benam K. H. Villenave R. Lucchesi C. Varone A. Hubeau C. Lee H.-H. (2016a). Small airway-on-a-chip enables analysis of human lung inflammation and drug responses *in vitro* . Nat. Methods 13 (2), 151–157. 10.1038/nmeth.3697 26689262

[B22] Bhatia S. N. Ingber D. E. (2014). Microfluidic organs-on-chips. Nat. Biotechnol. 32 (8), 760–772. 10.1038/nbt.2989 25093883

[B23] Bircsak K. M. DeBiasio R. Miedel M. Alsebahi A. Reddinger R. Saleh A. (2021). A 3D microfluidic liver model for high throughput compound toxicity screening in the OrganoPlate. Toxicology 450, 152667. 10.1016/j.tox.2020.152667 33359578

[B24] Blin A. Le Goff A. Magniez A. Poirault-Chassac S. Teste B. Sicot G. (2016). Microfluidic model of the platelet-generating organ: Beyond bone marrow biomimetics. Sci. Rep. 6 (1), 21700. 10.1038/srep21700 26898346 PMC4761988

[B25] Boeri L. Perottoni S. Izzo L. Giordano C. Albani D. (2021). Microbiota-host immunity communication in neurodegenerative disorders: Bioengineering challenges for *in vitro* modeling. Adv. Healthc. Mater. 10 (7), 2002043. 10.1002/adhm.202002043 PMC11468246 33661580

[B26] Busek M. Aizenshtadt A. Amirola-Martinez M. Delon L. Krauss S. (2022). Academic user view: Organ-on-a-Chip technology. Biosensors 12, 126. 10.3390/bios12020126 35200386 PMC8869899

[B27] Caballero D. Kaushik S. Correlo V. M. Oliveira J. M. Reis R. L. Kundu S. C. (2017). Organ-on-chip models of cancer metastasis for future personalized medicine: From chip to the patient. Biomaterials 149, 98–115. 10.1016/j.biomaterials.2017.10.005 29024838

[B28] Carvalho V. Gonçalves I. Lage T. Rodrigues R. O. Minas G. Teixeira S. F. C. F. (2021). 3D printing techniques and their applications to organ-on-a-chip platforms: A systematic review. Sensors 21, 3304. 10.3390/s21093304 34068811 PMC8126238

[B29] Chen J. Tang M. Xu D. (2021b). Integrated microfluidic chip coupled to mass spectrometry: A minireview of chip pretreatment methods and applications. J. Chromatogr. Open 1, 100021. 10.1016/j.jcoa.2021.100021

[B30] Chen M. B. Srigunapalan S. Wheeler A. R. Simmons C. A. (2013). A 3D microfluidic platform incorporating methacrylated gelatin hydrogels to study physiological cardiovascular cell–cell interactions. Lab a Chip 13 (13), 2591–2598. 10.1039/c3lc00051f 23525275

[B31] Chen J. Zhang Y. S. Zhang X. Liu C. (2021a). Organ-on-a-chip platforms for accelerating the evaluation of nanomedicine. Bioact. Mater. 6 (4), 1012–1027. 10.1016/j.bioactmat.2020.09.022 33102943 PMC7566214

[B32] Chethikkattuveli Salih A. R. Asif A. Samantasinghar A. Umer Farooqi H. M. Kim S. Choi K. H. (2022). Renal hypoxic reperfusion injury-on-chip model for studying combinational vitamin therapy. ACS Biomaterials Sci. Eng. 8 (9), 3733–3740. 10.1021/acsbiomaterials.2c00180 35878885

[B33] Chethikkattuveli Salih A. R. Hyun K. Asif A. Soomro A. M. Farooqi H. M. U. Kim Y. S. (2021). Extracellular matrix optimization for enhanced physiological relevance in hepatic tissue-chips. Polym. (Basel) 13 (17), 3016. 10.3390/polym13173016 PMC8434375 34503056

[B34] Cho M. Park J.-K. (2021). Modular 3D *in vitro* artery-mimicking multichannel system for recapitulating vascular stenosis and inflammation. Micromachines 12, 1528. 10.3390/mi12121528 34945377 PMC8709401

[B35] Cho Y. Na K. Jun Y. Won J. Yang J. H. Chung S. (2021). Three-dimensional *in vitro* lymphangiogenesis model in tumor microenvironment. Front. Bioeng. Biotechnol. 9, 697657. 10.3389/fbioe.2021.697657 34671596 PMC8520924

[B196] Choi J.-S. Meghani N. (2016). Impact of surface modification in BSA nanoparticles for uptake in cancer cells. Colloids Surf. B. 145, 653–661. 10.1016/j.colsurfb.2016.05.050 27289306

[B36] Chou D. B. Frismantas V. Milton Y. David R. Pop-Damkov P. Ferguson D. (2020). On-chip recapitulation of clinical bone marrow toxicities and patient-specific pathophysiology. Nat. Biomed. Eng. 4 (4), 394–406. 10.1038/s41551-019-0495-z 31988457 PMC7160021

[B37] Cong Y. Han X. Wang Y. Chen Z. Lu Y. Liu T. (2020). Drug toxicity evaluation based on organ-on-a-chip technology: A review. Micromachines (Basel) 11 (4), 381. 10.3390/mi11040381 32260191 PMC7230535

[B38] Convery N. Samardzhieva I. Stormonth-Darling J. M. Harrison S. Sullivan G. J. Gadegaard N. (2021). 3D printed tooling for injection molded microfluidics. Macromol. Mater. Eng. 306 (11), 2100464. 10.1002/mame.202100464

[B39] Costa L. Reis R. L. Silva-Correia J. Oliveira J. M. (2020). Microfluidics for angiogenesis research. Adv. Exp. Med. Biol. 1230, 97–119. 10.1007/978-3-030-36588-2_7 32285367

[B40] Cunningham T. J. Duester G. (2015). Mechanisms of retinoic acid signalling and its roles in organ and limb development. Nat. Rev. Mol. Cell Biol. 16 (2), 110–123. 10.1038/nrm3932 25560970 PMC4636111

[B41] Dér A. (2021). Special issue on versatile organ-on-a-chip devices. Micromachines 12, 1444. 10.3390/mi12121444 34945294 PMC8707123

[B42] Ehrlich A. Tsytkin-Kirschenzweig S. Ioannidis K. Ayyash M. Riu A. Note R. (2018). Microphysiological flux balance platform unravels the dynamics of drug induced steatosis. Lab a Chip 18 (17), 2510–2522. 10.1039/c8lc00357b PMC7004819 29992215

[B43] Ewart L. Apostolou A. Briggs S. A. Carman C. V. Chaff J. T. Heng A. R. (2021). Qualifying a human Liver-Chip for predictive toxicology: Performance assessment and economic implications. bioRxiv 2021, 472674. 10.1101/2021.12.14.472674

[B44] Ewart L. Apostolou A. Briggs S. A. Carman C. V. Chaff J. T. Heng A. R. (2022). Performance assessment and economic analysis of a human Liver-Chip for predictive toxicology. Commun. Med. 2 (1), 154. 10.1038/s43856-022-00209-1 36473994 PMC9727064

[B45] Farooqi H. M. Sammantasinghar A. Kausar F. Farooqi M. A. Chethikkattuveli Salih A. R. Hyun K. (2022). Study of the anticancer potential of plant extracts using liver tumor microphysiological system. Life 12, 135. 10.3390/life12020135 35207423 PMC8880716

[B46] Farooqi H. M. U. Kang B. Khalid M. A. U. Salih A. R. C. Hyun K. Park S. H. (2021). Real-time monitoring of liver fibrosis through embedded sensors in a microphysiological system. Nano Converg. 8 (1), 3. 10.1186/s40580-021-00253-y 33528697 PMC7855143

[B47] Fathi P. Holland G. Pan D. Esch M. B. (2020). Lymphatic vessel on a chip with capability for exposure to cyclic fluidic flow. ACS Appl. Bio Mater. 3 (10), 6697–6707. 10.1021/acsabm.0c00609 PMC13218302 35019335

[B48] Ferreira D. A. Rothbauer M. Conde J. P. Ertl P. Oliveira C. Granja P. L. (2021). A fast alternative to soft lithography for the fabrication of organ-on-a-chip elastomeric-based devices and microactuators. Adv. Sci. 8 (8), 2003273. 10.1002/advs.202003273 PMC8061392 33898174

[B49] França C. M. Tahayeri A. Rodrigues N. S. Ferdosian S. Puppin Rontani R. M. Sereda G. (2020). The tooth on-a-chip: A microphysiologic model system mimicking the biologic interface of the tooth with biomaterials. Lab a Chip 20 (2), 405–413. 10.1039/c9lc00915a PMC7395925 31854401

[B50] Freag M. S. Namgung B. Reyna Fernandez M. E. Gherardi E. Sengupta S. Jang H. L. (2021). Human nonalcoholic steatohepatitis on a chip. Hepatol. Commun. 5 (2), 217–233. 10.1002/hep4.1647 33553970 PMC7850303

[B51] Fritschen A. Blaeser A. (2021). Biosynthetic, biomimetic, and self-assembled vascularized Organ-on-a-Chip systems. Biomaterials 268, 120556. 10.1016/j.biomaterials.2020.120556 33310539

[B52] Garcia-Gutierrez E. Cotter P. D. (2022). Relevance of organ(s)-on-a-chip systems to the investigation of food-gut microbiota-host interactions. Crit. Rev. Microbiol. 48 (4), 463–488. 10.1080/1040841X.2021.1979933 34591726

[B53] Gard A. L. Luu R. J. Miller C. R. Maloney R. Cain B. P. Marr E. E. (2021). High-throughput human primary cell-based airway model for evaluating influenza, coronavirus, or other respiratory viruses *in vitro* . Sci. Rep. 11 (1), 14961. 10.1038/s41598-021-94095-7 34294757 PMC8298517

[B54] Glieberman A. L. Pope B. D. Zimmerman J. F. Liu Q. Ferrier J. P. Kenty J. H. R. (2019). Synchronized stimulation and continuous insulin sensing in a microfluidic human Islet on a Chip designed for scalable manufacturing. Lab a Chip 19 (18), 2993–3010. 10.1039/c9lc00253g PMC6814249 31464325

[B55] Görgens C. Ramme A. P. Guddat S. Schrader Y. Winter A. Dehne E.-M. (2021). Organ-on-a-chip: Determine feasibility of a human liver microphysiological model to assess long-term steroid metabolites in sports drug testing. Drug Test. Analysis 13 (11-12), 1921–1928. 10.1002/dta.3161 34505743

[B56] Gough A. Soto-Gutierrez A. Vernetti L. Ebrahimkhani M. R. Stern A. M. Taylor D. L. (2021). Human biomimetic liver microphysiology systems in drug development and precision medicine. Nat. Rev. Gastroenterology hepatology 18 (4), 252–268. 10.1038/s41575-020-00386-1 33335282 PMC9106093

[B57] Guo S.-C. Tao S.-C. Dawn H. (2018). Microfluidics-based on-a-chip systems for isolating and analysing extracellular vesicles. J. Extracell. Vesicles 7 (1), 1508271. 10.1080/20013078.2018.1508271 30151077 PMC6104604

[B58] Guo Y. Luo R. Wang Y. Deng P. Song T. Zhang M. (2021). SARS-CoV-2 induced intestinal responses with a biomimetic human gut-on-chip. Sci. Bull. 66 (8), 783–793. 10.1016/j.scib.2020.11.015 PMC7704334 33282445

[B59] Guttenplan A. P. M. Tahmasebi Birgani Z. Giselbrecht S. Truckenmüller R. K. Habibović P. (2021). Chips for biomaterials and biomaterials for chips: Recent advances at the interface between microfabrication and biomaterials research. Adv. Healthc. Mater. 10 (14), 2100371. 10.1002/adhm.202100371 PMC11468311 34033239

[B60] Hachey S. J. Movsesyan S. Nguyen Q. H. Burton-Sojo G. Tankazyan A. Wu J. (2021). An *in vitro* vascularized micro-tumor model of human colorectal cancer recapitulates *in vivo* responses to standard-of-care therapy. Lab a Chip 21 (7), 1333–1351. 10.1039/d0lc01216e PMC8525497 33605955

[B61] Han C. Labuz J. M. Takayama S. Park J. (2014). “Chapter 20 - organs-on-a-chip,” in Tissue engineering. Editors Blitterswijk C. A. V. De Boer J. Second Edition (Oxford: Academic Press), 717–746.

[B62] Han J. J. (2023). FDA Modernization Act 2.0 allows for alternatives to animal testing. Artif. Organs 47 (3), 449–450. 10.1111/aor.14503 36762462

[B63] Hansen F. A. Petersen N. J. Kutter J. P. Pedersen-Bjergaard S. (2022). Electromembrane extraction in microfluidic formats. J. Sep. Sci. 45 (1), 246–257. 10.1002/jssc.202100603 34562339

[B64] Hassell B. A. Goyal G. Lee E. Sontheimer-Phelps A. Levy O. Chen C. S. (2017). Human organ chip models recapitulate orthotopic lung cancer growth, therapeutic responses, and tumor dormancy *in vitro* . Cell Rep. 21 (2), 508–516. 10.1016/j.celrep.2017.09.043 29020635

[B65] Hassell B. A. Goyal G. Lee E. Sontheimer-Phelps A. Levy O. Chen C. S. (2018). Human organ chip models recapitulate orthotopic lung cancer growth, therapeutic responses, and tumor dormancy *in vitro* . Cell Rep. 23 (12), 3698. 10.1016/j.celrep.2018.06.028 29925009

[B66] Herland A. Maoz B. M. Das D. Somayaji M. R. Prantil-Baun R. Novak R. (2020). Quantitative prediction of human pharmacokinetic responses to drugs via fluidically coupled vascularized organ chips. Nat. Biomed. Eng. 4 (4), 421–436. 10.1038/s41551-019-0498-9 31988459 PMC8011576

[B67] Holloway P. M. Willaime-Morawek S. Siow R. Barber M. Owens R. M. Sharma A. D. (2021). Advances in microfluidic *in vitro* systems for neurological disease modeling. J. Neurosci. Res. 99 (5), 1276–1307. 10.1002/jnr.24794 33583054

[B68] Homan K. A. Kolesky D. B. Skylar-Scott M. A. Herrmann J. Obuobi H. Moisan A. (2016). Bioprinting of 3D convoluted renal proximal tubules on perfusable chips. Sci. Rep. 6 (1), 34845. 10.1038/srep34845 27725720 PMC5057112

[B69] Hoogduijn M. J. Montserrat N. van der Laan L. J. W. Dazzi F. Perico N. Kastrup J. (2020). The emergence of regenerative medicine in organ transplantation: 1st European cell therapy and organ regeneration section meeting. Transpl. Int. 33 (8), 833–840. 10.1111/tri.13608 32237237 PMC7497223

[B70] House A. Atalla I. Lee E. J. Guvendiren M. (2021). Designing biomaterial platforms for cardiac tissue and disease modeling. Adv. NanoBiomed Res. 1 (1), 2000022. 10.1002/anbr.202000022 33709087 PMC7942203

[B71] Hübner J. Raschke M. Rütschle I. Gräßle S. Hasenberg T. Schirrmann K. (2018). Simultaneous evaluation of anti-EGFR-induced tumour and adverse skin effects in a microfluidic human 3D co-culture model. Sci. Rep. 8 (1), 15010. 10.1038/s41598-018-33462-3 30301942 PMC6177413

[B72] Huh D. Kim H. J. Fraser J. P. Shea D. E. Khan M. Bahinski A. (2013). Microfabrication of human organs-on-chips. Nat. Protoc. 8 (11), 2135–2157. 10.1038/nprot.2013.137 24113786

[B73] Huh D. Leslie D. C. Matthews B. D. Fraser J. P. Jurek S. Hamilton G. A. (2012). A human disease model of drug toxicity–induced pulmonary edema in a lung-on-a-chip microdevice. Sci. Transl. Med. 4 (159), 159ra147. 10.1126/scitranslmed.3004249 PMC8265389 23136042

[B74] Huh D. Matthews B. D. Mammoto A. Montoya-Zavala M. Hsin H. Y. Ingber D. E. (2010). Reconstituting organ-level lung functions on a chip. Science 328 (5986), 1662–1668. 10.1126/science.1188302 20576885 PMC8335790

[B75] Ingber D. E. (2022). Human organs-on-chips for disease modelling, drug development and personalized medicine. Nat. Rev. Genet. 23 (8), 467–491. 10.1038/s41576-022-00466-9 35338360 PMC8951665

[B76] Jahagirdar D. Bangde P. Jain R. Dandekar P. (2021). Degenerative disease-on-a-chip: Developing microfluidic models for rapid availability of newer therapies. Biotechnol. J. 16 (10), 2100154. 10.1002/biot.202100154 34390543

[B77] Jain A. Barrile R. van der Meer A. D. Mammoto A. Mammoto T. De Ceunynck K. (2018). Primary human lung alveolus-on-a-chip model of intravascular thrombosis for assessment of therapeutics. Clin. Pharmacol. Ther. 103 (2), 332–340. 10.1002/cpt.742 28516446 PMC5693794

[B78] Jang K.-J. Mehr A. P. Hamilton G. A. McPartlin L. A. Chung S. Suh K.-Y. (2013). Human kidney proximal tubule-on-a-chip for drug transport and nephrotoxicity assessment. Integr. Biol. 5 (9), 1119–1129. 10.1039/c3ib40049b 23644926

[B79] Jang K.-J. Otieno M. A. Ronxhi J. Lim H.-K. Ewart L. Kodella K. R. (2019). Reproducing human and cross-species drug toxicities using a Liver-Chip. Sci. Transl. Med. 11 (517), eaax5516. 10.1126/scitranslmed.aax5516 31694927

[B80] Jin Z. Li Y. Yu K. Liu L. Fu J. Yao X. (2021). 3D printing of physical organ models: Recent developments and challenges. Adv. Sci. 8 (17), 2101394. 10.1002/advs.202101394 PMC8425903 34240580

[B81] Kang S. Park S. E. Huh D. D. (2021). Organ-on-a-chip technology for nanoparticle research. Nano Converg. 8 (1), 20. 10.1186/s40580-021-00270-x 34236537 PMC8266951

[B82] Kausar F. Farooqi M.-A. Farooqi H.-M.-U. Salih A.-R.-C. Khalil A.-A.-K. Kang C.-w. (2021). Phytochemical investigation, antimicrobial, antioxidant and anticancer activities of acer cappadocicum gled. Life 11, 656. 10.3390/life11070656 34357028 PMC8306863

[B83] Kausar F. Kim K.-H. Farooqi H. M. Farooqi M. A. Kaleem M. Waqar R. (2022). Evaluation of antimicrobial and anticancer activities of selected medicinal plants of himalayas, Pakistan. Plants 11, 48. 10.3390/plants11010048 PMC8747275 35009052

[B84] Kavand H. Nasiri R. Herland A. (2022). Advanced materials and sensors for microphysiological systems: Focus on electronic and electrooptical interfaces. Adv. Mater. 34 (17), 2107876. 10.1002/adma.202107876 34913206

[B85] Kengelbach-Weigand A. Thielen C. Bäuerle T. Götzl R. Gerber T. Körner C. (2021). Personalized medicine for reconstruction of critical-size bone defects – A translational approach with customizable vascularized bone tissue. npj Regen. Med. 6 (1), 49. 10.1038/s41536-021-00158-8 34413320 PMC8377075

[B86] Kerns S. J. Belgur C. Petropolis D. Kanellias M. Barrile R. Sam J. (2021). Human immunocompetent Organ-on-chip platforms allow safety profiling of tumor-targeted T-cell bispecific antibodies. eLife 10, e67106. 10.7554/eLife.67106 34378534 PMC8373379

[B87] Khalid M. A. U. Kim K. H. Chethikkattuveli Salih A. R. Hyun K. Park S. H. Kang B. (2022). High performance inkjet printed embedded electrochemical sensors for monitoring hypoxia in a gut bilayer microfluidic chip. Lab a Chip 22 (9), 1764–1778. 10.1039/d1lc01079d 35244110

[B192] Kim H. B. Meghani N. Park M. Lee S. H. Lee S. R. Cho Y.-J. (2020). Electrohydrodynamically atomized pH-responsive PLGA/ZnO quantum dots for local delivery in lung cancer. Macromol. Res. 28, 407–414. 10.1007/s13233-020-8053-9

[B88] Kim M. H. Lee K.-T. Lee J. S. Shin J. Cui B. Yang K. (2021b). Skin prick testing predicts peach hypersensitivity reactions. Nat. Biomed. Eng. 5 (8), 830–832. 10.4168/aair.2021.13.6.830 34734502 PMC8569024

[B89] Kim M. H. van Noort D. Sung J. H. Park S. (2021a). Organ-on-a-Chip for studying gut-brain interaction mediated by extracellular vesicles in the gut microenvironment. Int. J. Mol. Sci. 22, 13513. 10.3390/ijms222413513 34948310 PMC8707342

[B90] Kim M. H. Asif A. Chethikkattuveli Salih A. R. Lee J.-W. Hyun K.-N. Choi K.-H. (2021c). Gravity-based flow efficient perfusion culture system for spheroids mimicking liver inflammation. Biomedicines 9, 1369. 10.3390/biomedicines9101369 34680487 PMC8533112

[B91] Kujala V. J. Pasqualini F. S. Goss J. A. Nawroth J. C. Parker K. K. (2016). Laminar ventricular myocardium on a microelectrode array-based chip. J. Mater. Chem. B 4 (20), 3534–3543. 10.1039/c6tb00324a 32263387

[B92] Kurth F. Györvary E. Heub S. Ledroit D. Paoletti S. Renggli K. (2020). “Chapter 3 - organs-on-a-chip engineering,” in Organ-on-a-chip. Editors Hoeng J. Bovard D. Peitsch M. C. (Cambridge, Massachusetts, United States: Academic Press), 47–130.

[B93] Kutys M. L. Polacheck W. J. Welch M. K. Gagnon K. A. Koorman T. Kim S. (2020). Uncovering mutation-specific morphogenic phenotypes and paracrine-mediated vessel dysfunction in a biomimetic vascularized mammary duct platform. Nat. Commun. 11 (1), 3377. 10.1038/s41467-020-17102-x 32632100 PMC7338408

[B94] Lai B. F. L. Lu R. X. Z. Davenport Huyer L. Kakinoki S. Yazbeck J. Wang E. Y. (2021). A well plate–based multiplexed platform for incorporation of organoids into an organ-on-a-chip system with a perfusable vasculature. Nat. Protoc. 16 (4), 2158–2189. 10.1038/s41596-020-00490-1 33790475

[B95] Lam S. F. Bishop K. W. Mintz R. Fang L. Achilefu S. (2021). Calcium carbonate nanoparticles stimulate cancer cell reprogramming to suppress tumor growth and invasion in an organ-on-a-chip system. Sci. Rep. 11 (1), 9246. 10.1038/s41598-021-88687-6 33927272 PMC8084943

[B96] Lee P. J. Hung P. J. Lee L. P. (2007). An artificial liver sinusoid with a microfluidic endothelial-like barrier for primary hepatocyte culture. Biotechnol. Bioeng. 97 (5), 1340–1346. 10.1002/bit.21360 17286266

[B97] Lee-Montiel F. T. Laemmle A. Charwat V. Dumont L. Lee C. S. Huebsch N. (2021). Integrated isogenic human induced pluripotent stem cell–based liver and heart microphysiological systems predict unsafe drug–drug interaction. Front. Pharmacol. 12, 667010. 10.3389/fphar.2021.667010 34025426 PMC8138446

[B98] Li K. Yang X. Xue C. Zhao L. Zhang Y. Gao X. (2019). Biomimetic human lung-on-a-chip for modeling disease investigation. Biomicrofluidics 13 (3), 031501. 10.1063/1.5100070 31263514 PMC6597342

[B99] Lin N. Y. C. Homan K. A. Robinson S. S. Kolesky D. B. Duarte N. Moisan A. (2019). Renal reabsorption in 3D vascularized proximal tubule models. Proc. Natl. Acad. Sci. 116 (12), 5399–5404. 10.1073/pnas.1815208116 30833403 PMC6431199

[B100] Lin Q. Lim J. Y. C. Xue K. Yew P. Y. M. Owh C. Chee P. L. (2020). Sanitizing agents for virus inactivation and disinfection. VIEW 1 (2), e16. 10.1002/viw2.16 34766164 PMC7267133

[B101] Lingadahalli Kotreshappa S. Nayak C. G. Krishnan Venkata S. (2023). A review on the role of microflow parameter measurements for microfluidics applications. Systems 391, 1485–1498. 10.1007/s00216-007-1827-5

[B102] Liu H. Bolonduro O. A. Hu N. Ju J. Rao A. A. Duffy B. M. (2020). Heart-on-a-Chip model with integrated extra- and intracellular bioelectronics for monitoring cardiac electrophysiology under acute hypoxia. Nano Lett. 20 (4), 2585–2593. 10.1021/acs.nanolett.0c00076 32092276

[B103] Liu X. Fang J. Huang S. Wu X. Xie X. Wang J. (2021). Tumor-on-a-chip: From bioinspired design to biomedical application. Microsystems Nanoeng. 7 (1), 50. 10.1038/s41378-021-00277-8 PMC8433302 34567763

[B104] Liu X. Sato N. Shimosato Y. Wang T.-W. Denda T. Chang Y.-H. (2022a). CHIP-associated mutant ASXL1 in blood cells promotes solid tumor progression. Cancer Sci. 113 (4), 1182–1194. 10.1111/cas.15294 35133065 PMC8990791

[B105] Liu X. Su Q. Zhang X. Yang W. Ning J. Jia K. (2022b). Recent advances of organ-on-a-chip in cancer modeling research. Biosensors 12, 1045. 10.3390/bios12111045 36421163 PMC9688857

[B106] Low L. A. Mummery C. Berridge B. R. Austin C. P. Tagle D. A. (2021). Organs-on-chips: Into the next decade. Nat. Rev. Drug Discov. 20 (5), 345–361. 10.1038/s41573-020-0079-3 32913334

[B107] Lu R. X. Z. Radisic M. (2021). Organ-on-a-chip platforms for evaluation of environmental nanoparticle toxicity. Bioact. Mater. 6 (9), 2801–2819. 10.1016/j.bioactmat.2021.01.021 33665510 PMC7900603

[B108] Manafi N. Shokri F. Achberger K. Hirayama M. Mohammadi M. H. Noorizadeh F. (2021). Organoids and organ chips in ophthalmology. Ocular Surf. 19, 1–15. 10.1016/j.jtos.2020.11.004 33220469

[B109] Mansoorifar A. Gordon R. Bergan R. C. Bertassoni L. E. (2021). Bone-on-a-Chip: Microfluidic technologies and microphysiologic models of bone tissue. Adv. Funct. Mater. 31 (6), 2006796. 10.1002/adfm.202006796 35422682 PMC9007546

[B110] Maoz B. M. Herland A. FitzGerald E. A. Grevesse T. Vidoudez C. Pacheco A. R. (2018). A linked organ-on-chip model of the human neurovascular unit reveals the metabolic coupling of endothelial and neuronal cells. Nat. Biotechnol. 36 (9), 865–874. 10.1038/nbt.4226 30125269 PMC9254231

[B111] Martinez E. St-Pierre J.-P. Variola F. (2019). Advanced bioengineering technologies for preclinical research. Adv. Phys. X 4 (1), 1622451. 10.1080/23746149.2019.1622451

[B112] Maschmeyer I. Kakava S. (2022). Organ-on-a-Chip. Adv. Biochem. engineering/biotechnology 179, 311–342. 10.1007/10_2020_135 32948885

[B113] Mastrangeli M. Millet S. Orchid Partners T. Van den Eijnden-van Raaij J. (2019). Organ-on-chip in development: Towards a roadmap for organs-on-chip. Altex 36 (4), 650–668. 10.14573/altex.1908271 31664458

[B114] McGonigle P. Ruggeri B. (2014). Animal models of human disease: Challenges in enabling translation. Biochem. Pharmacol. 87 (1), 162–171. 10.1016/j.bcp.2013.08.006 23954708

[B190] Meghani N. Kim K. H. Kim S. H. Lee S. H. Choi K. H. (2020). Evaluation and live monitoring of pH-responsive HSA-ZnO nanoparticles using a lung-on-a-chip model. Arch. Pharm. Res. 43 (5), 503–513. 10.1007/s12272-020-01236-z 32472315

[B197] Meghani N. M. Amin H. H. Park C. Park J. B. Cui J. H. Cao Q. R. (2018). Design and evaluation of clickable gelatin-oleic nanoparticles using fattigation-platform for cancer therapy. Int. J. Pharm. 545 (1–2), 101–112. 10.1016/j.ijpharm.2018.04.047 29698822

[B194] Meghani N. M. Amin H. H. Lee B. J. (2017). Mechanistic applications of click chemistry for pharmaceutical drug discovery and drug delivery. Drug. Discov. Today. 22 (11), 1604–1619. 10.1016/j.drudis.2017.07.007 28754291

[B115] Mencattini A. Mattei F. Schiavoni G. Gerardino A. Businaro L. Di Natale C. (2019). From petri dishes to organ on chip platform: The increasing importance of machine learning and image analysis. Front. Pharmacol. 10, 100. 10.3389/fphar.2019.00100 30863306 PMC6399655

[B116] Miccoli B. Braeken D. Li Y. E. (2018). Brain-on-a-chip devices for drug screening and disease modeling applications. Curr. Pharm. Des. 24 (45), 5419–5436. 10.2174/1381612825666190220161254 30806304

[B117] Miller C. P. Shin W. Ahn E. H. Kim H. J. Kim D.-H. (2020). Engineering microphysiological immune system responses on chips. Trends Biotechnol. 38 (8), 857–872. 10.1016/j.tibtech.2020.01.003 32673588 PMC7368088

[B118] Mondadori C. Palombella S. Salehi S. Talò G. Visone R. Rasponi M. (2021). Recapitulating monocyte extravasation to the synovium in an organotypic microfluidic model of the articular joint. Biofabrication 13 (4), 045001. 10.1088/1758-5090/ac0c5e 34139683

[B119] Morelli M. Kurek D. Ng C. P. Queiroz K. (2023). Gut-on-a-Chip models: Current and future perspectives for host–microbial interactions research. Biomedicines 11, 619. 10.3390/biomedicines11020619 36831155 PMC9953162

[B120] Mosavati B. Oleinikov A. V. Du E. (2020). Development of an organ-on-a-chip-device for study of placental pathologies. Int. J. Mol. Sci. 21 (22), 8755. 10.3390/ijms21228755 33228194 PMC7699553

[B121] Musah S. Mammoto A. Ferrante T. C. Jeanty S. S. F. Hirano-Kobayashi M. Mammoto T. (2017). Mature induced-pluripotent-stem-cell-derived human podocytes reconstitute kidney glomerular-capillary-wall function on a chip. Nat. Biomed. Eng. 1 (5), 0069. 10.1038/s41551-017-0069 29038743 PMC5639718

[B122] Naik S. Wood A. R. Ongenaert M. Saidiyan P. Elstak E. D. Lanz H. L. (2021). A 3D renal proximal tubule on chip model phenocopies Lowe syndrome and dent II disease tubulopathy. Int. J. Mol. Sci. 22, 5361. 10.3390/ijms22105361 34069732 PMC8161077

[B123] Nawroth J. C. Lucchesi C. Cheng D. Shukla A. Ngyuen J. Shroff T. (2020). A microengineered airway lung chip models key features of viral-induced exacerbation of asthma. Am. J. Respir. Cell Mol. Biol. 63 (5), 591–600. 10.1165/rcmb.2020-0010MA 32706623

[B124] Nawroth J. C. Petropolis D. B. Manatakis D. V. Maulana T. I. Burchett G. Schlünder K. (2021). Modeling alcohol-associated liver disease in a human Liver-Chip. Cell Rep. 36 (3), 109393. 10.1016/j.celrep.2021.109393 34289365 PMC8342038

[B125] Nelson M. T. Charbonneau M. R. Coia H. G. Castillo M. J. Holt C. Greenwood E. S. (2021). Characterization of an engineered live bacterial therapeutic for the treatment of phenylketonuria in a human gut-on-a-chip. Nat. Commun. 12 (1), 2805. 10.1038/s41467-021-23072-5 33990606 PMC8121789

[B126] Nitsche K. S. Müller I. Malcomber S. Carmichael P. L. Bouwmeester H. (2022). Implementing organ-on-chip in a next-generation risk assessment of chemicals: A review. Archives Toxicol. 96 (3), 711–741. 10.1007/s00204-022-03234-0 PMC8850248 35103818

[B199] Novak R. Ingram M. Clauson S. Das D. Delahanty A. Herland A. (2019). A robotic platform for fluidically-linked human body-on-chips experimentation. BioRxiv. 10.1101/569541

[B127] Ortega-Prieto A. M. Skelton J. K. Wai S. N. Large E. Lussignol M. Vizcay-Barrena G. (2018). 3D microfluidic liver cultures as a physiological preclinical tool for Hepatitis B virus infection. Nat. Commun. 9 (1), 682. 10.1038/s41467-018-02969-8 29445209 PMC5813240

[B128] Paek J. Park S. E. Lu Q. Park K.-T. Cho M. Oh J. M. (2019). Microphysiological engineering of self-assembled and perfusable microvascular beds for the production of vascularized three-dimensional human microtissues. ACS Nano 13 (7), 7627–7643. 10.1021/acsnano.9b00686 31194909

[B195] Park C. Meghani N. Amin H. Tran P. H.-L. Tran T. T.-D. Nguyen V. H. (2019). The roles of short and long chain fatty acids on physicochemical properties and improved cancer targeting of albumin-based fattigation-platform nanoparticles containing doxorubicin. Int. J. Pharm. 564, 124–135. 10.1016/j.ijpharm.2019.04.038 30991133

[B129] Peck R. W. Hinojosa C. D. Hamilton G. A. (2020). Organs-on-Chips in clinical Pharmacology: Putting the patient into the center of treatment selection and drug development. Clin. Pharmacol. Ther. 107 (1), 181–185. 10.1002/cpt.1688 31758803 PMC6977308

[B130] Pediaditakis I. Kodella K. R. Manatakis D. V. Le C. Y. Hinojosa C. D. Tien-Street W. (2021). Modeling alpha-synuclein pathology in a human brain-chip to assess blood-brain barrier disruption. Nat. Commun. 12 (1), 5907. 10.1038/s41467-021-26066-5 34625559 PMC8501050

[B131] Petrosyan A. Cravedi P. Villani V. Angeletti A. Manrique J. Renieri A. (2019). A glomerulus-on-a-chip to recapitulate the human glomerular filtration barrier. Nat. Commun. 10 (1), 3656. 10.1038/s41467-019-11577-z 31409793 PMC6692336

[B132] Picollet-D’hahan N. Zuchowska A. Lemeunier I. Le Gac S. (2021). Multiorgan-on-a-Chip: A systemic approach to model and decipher inter-organ communication. Trends Biotechnol. 39 (8), 788–810. 10.1016/j.tibtech.2020.11.014 33541718

[B133] Plebani R. Potla R. Soong M. Bai H. Izadifar Z. Jiang A. (2022). Modeling pulmonary cystic fibrosis in a human lung airway-on-a-chip. J. Cyst. Fibros. 21 (4), 606–615. 10.1016/j.jcf.2021.10.004 34799298

[B134] Polini A. Moroni L. (2021). The convergence of high-tech emerging technologies into the next stage of organ-on-a-chips. Biomaterials Biosyst. 1, 100012. 10.1016/j.bbiosy.2021.100012 PMC9934418 36825163

[B135] Preetam S. Nahak B. K. Patra S. Toncu D. C. Park S. Syväjärvi M. (2022). Emergence of microfluidics for next generation biomedical devices. Biosens. Bioelectron. X 10, 100106. 10.1016/j.biosx.2022.100106

[B136] Ragelle H. Dernick K. Khemais S. Keppler C. Cousin L. Farouz Y. (2020). Human retinal microvasculature-on-a-chip for drug discovery. Adv. Healthc. Mater. 9 (21), 2001531. 10.1002/adhm.202001531 32975047

[B137] Ramadan Q. Zourob M. (2020). Organ-on-a-chip engineering: Toward bridging the gap between lab and industry. Biomicrofluidics 14 (4), 041501. 10.1063/5.0011583 32699563 PMC7367691

[B138] Ratri M. C. Brilian A. I. Setiawati A. Nguyen H. T. Soum V. Shin K. (2021). Recent advances in regenerative tissue fabrication: Tools, materials, and microenvironment in hierarchical aspects. Adv. NanoBiomed Res. 1 (5), 2000088. 10.1002/anbr.202000088

[B139] Regmi S. Poudel C. Adhikari R. Luo K. Q. (2022). Applications of microfluidics and organ-on-a-chip in cancer research. Biosensors 12, 459. 10.3390/bios12070459 35884262 PMC9313151

[B140] Ribas J. Zhang Y. S. Pitrez P. R. Leijten J. Miscuglio M. Rouwkema J. (2017). Biomechanical strain exacerbates inflammation on a progeria-on-a-chip model. Small 13 (15), 1603737. 10.1002/smll.201603737 PMC5545787 28211642

[B141] Rogal J. Binder C. Kromidas E. Roosz J. Probst C. Schneider S. (2020). WAT-on-a-chip integrating human mature white adipocytes for mechanistic research and pharmaceutical applications. Sci. Rep. 10 (1), 6666. 10.1038/s41598-020-63710-4 32313039 PMC7170869

[B142] Rokhsar Talabazar F. Jafarpour M. Zuvin M. Chen H. Gevari M. T. Villanueva L. G. (2021). Design and fabrication of a vigorous “cavitation-on-a-chip” device with a multiple microchannel configuration. Microsystems Nanoeng. 7 (1), 44. 10.1038/s41378-021-00270-1 PMC8433160 34567757

[B198] Ronaldson-Bouchard K. Teles D. Yeager K. Tavakol D. N. Zhao Y. Chramiec A. (2022). A multi-organ chip with matured tissue niches linked by vascular flow. Nat. Biomed. Eng. 6, 351–371. 10.1038/s41551-022-00882-6 35478225 PMC9250010

[B143] Rowe C. Shaeri M. Large E. Cornforth T. Robinson A. Kostrzewski T. (2018). Perfused human hepatocyte microtissues identify reactive metabolite-forming and mitochondria-perturbing hepatotoxins. Toxicol Vitro 46, 29–38. 10.1016/j.tiv.2017.09.012 28919358

[B144] Roye Y. Bhattacharya R. Mou X. Zhou Y. Burt M. A. Musah S. (2021). A personalized glomerulus chip engineered from stem cell-derived epithelium and vascular endothelium. Micromachines 12, 967. 10.3390/mi12080967 34442589 PMC8400556

[B145] Ryu J. Yu H. Kang D. Kim T. Kim J. (2021). Microfluidic valvular chips and a numerical lymphatic vessel model for the study of lymph transport characteristics. Lab a Chip 21 (11), 2283–2293. 10.1039/d1lc00022e 33942040

[B146] Salih A. R. C. Farooqi H. M. U. Kim Y. S. Lee S. H. Choi K. H. (2020). Impact of serum concentration in cell culture media on tight junction proteins within a multiorgan microphysiological system. Microelectron. Eng. 232, 111405. 10.1016/j.mee.2020.111405

[B147] Samantasinghar A. Sunildutt N. P. Ahmed F. Soomro A. M. Salih A. R. C. Parihar P. (2023). A comprehensive review of key factors affecting the efficacy of antibody drug conjugate. Biomed. Pharmacother. 161, 114408. 10.1016/j.biopha.2023.114408 36841027

[B148] Santoso J. W. McCain M. L. (2020). Neuromuscular disease modeling on a chip. Dis. models Mech. 13 (7), dmm044867. 10.1242/dmm.044867 PMC7358135 32817118

[B149] Sasserath T. Rumsey J. W. McAleer C. W. Bridges L. R. Long C. J. Elbrecht D. (2020). Differential monocyte actuation in a three-organ functional innate immune system-on-a-chip. Adv. Sci. 7 (13), 2000323. 10.1002/advs.202000323 PMC7341107 32670763

[B150] Schuster B. Junkin M. Kashaf S. S. Romero-Calvo I. Kirby K. Matthews J. (2020). Automated microfluidic platform for dynamic and combinatorial drug screening of tumor organoids. Nat. Commun. 11 (1), 5271. 10.1038/s41467-020-19058-4 33077832 PMC7573629

[B151] Semertzidou A. Brosens J. J. McNeish I. Kyrgiou M. (2020). Organoid models in gynaecological oncology research. Cancer Treat. Rev. 90, 102103. 10.1016/j.ctrv.2020.102103 32932156

[B152] Shanti A. Hallfors N. Petroianu G. A. Planelles L. Stefanini C. (2021). Lymph nodes-on-chip: Promising immune platforms for pharmacological and toxicological applications. Front. Pharmacol. 12, 711307. 10.3389/fphar.2021.711307 34483920 PMC8415712

[B153] Si L. Bai H. Oh C. Y. Zhang T. Hong F. Jiang A. (2021b). Self-assembling short immunostimulatory duplex RNAs with broad spectrum antiviral activity. bioRxiv 19, 469183. 10.1101/2021.11.19.469183 PMC9398551 36032397

[B154] Si L. Bai H. Rodas M. Cao W. Oh C. Y. Jiang A. (2021a). A human-airway-on-a-chip for the rapid identification of candidate antiviral therapeutics and prophylactics. Nat. Biomed. Eng. 5 (8), 815–829. 10.1038/s41551-021-00718-9 33941899 PMC8387338

[B155] Silvani G. Basirun C. Wu H. Mehner C. Poole K. Bradbury P. (2021). A 3D-bioprinted vascularized glioblastoma-on-a-chip for studying the impact of simulated microgravity as a novel pre-clinical approach in brain tumor therapy. Adv. Ther. 4 (11), 2100106. 10.1002/adtp.202100106

[B156] Sitti M. Wiersma D. S. (2020). Pros and cons: Magnetic versus optical microrobots. Adv. Mater. 32 (20), 1906766. 10.1002/adma.201906766 32053227

[B157] Skardal A. Shupe T. Atala A. (2016). Organoid-on-a-chip and body-on-a-chip systems for drug screening and disease modeling. Drug Discov. today 21 (9), 1399–1411. 10.1016/j.drudis.2016.07.003 27422270 PMC9039871

[B158] Sontheimer-Phelps A. Hassell B. A. Ingber D. E. (2019). Modelling cancer in microfluidic human organs-on-chips. Nat. Rev. Cancer 19 (2), 65–81. 10.1038/s41568-018-0104-6 30647431

[B159] Soomro A. M. Jawed B. Soomro J. B. Ahmed Ansari J. Ahmed F. Waqas M. (2022). Flexible fluidic-type strain sensors for wearable and robotic applications fabricated with novel conductive liquids: A review. Electronics 11, 2903. 10.3390/electronics11182903

[B160] Spijkers X. M. Pasteuning-Vuhman S. Dorleijn J. C. Vulto P. Wevers N. R. Pasterkamp R. J. (2021). A directional 3D neurite outgrowth model for studying motor axon biology and disease. Sci. Rep. 11 (1), 2080. 10.1038/s41598-021-81335-z 33483540 PMC7822896

[B161] Stengelin E. Thiele J. Seiffert S. (2022). Multiparametric material functionality of microtissue-based *in vitro* models as alternatives to animal testing. Adv. Sci. 9 (10), 2105319. 10.1002/advs.202105319 PMC8981905 35043598

[B162] Strelez C. Chilakala S. Ghaffarian K. Lau R. Spiller E. Ung N. (2021). Human colorectal cancer-on-chip model to study the microenvironmental influence on early metastatic spread. iScience 24 (5), 102509. 10.1016/j.isci.2021.102509 34113836 PMC8169959

[B163] Stucki J. D. Hobi N. Galimov A. Stucki A. O. Schneider-Daum N. Lehr C.-M. (2018). Medium throughput breathing human primary cell alveolus-on-chip model. Sci. Rep. 8 (1), 14359. 10.1038/s41598-018-32523-x 30254327 PMC6156575

[B164] Sun H. Jia Y. Dong H. Dong D. Zheng J. (2020). Combining additive manufacturing with microfluidics: An emerging method for developing novel organs-on-chips. Curr. Opin. Chem. Eng. 28, 1–9. 10.1016/j.coche.2019.10.006

[B165] Sung J. H. (2021). Multi-organ-on-a-chip for pharmacokinetics and toxicokinetic study of drugs. Expert Opin. drug metabolism Toxicol. 17 (8), 969–986. 10.1080/17425255.2021.1908996 33764248

[B166] Tovar-Lopez F. Thurgood P. Gilliam C. Nguyen N. Pirogova E. Khoshmanesh K. (2019). A microfluidic system for studying the effects of disturbed flow on endothelial cells. Front. Bioeng. Biotechnol. 7, 81. 10.3389/fbioe.2019.00081 31111027 PMC6499196

[B191] Valverde M. G. Faria J. Sendino Garví E. Janssen M. J. Masereeuw R. Mihăilă S. M. (2022). Organs-on-chip technology: A tool to tackle genetic kidney diseases. Pediatr. Nephrol. 37 (12), 2985–2996. 10.1007/s00467-022-05508-2 35286457 PMC9587109

[B167] van Berlo D. van de Steeg E. Amirabadi H. E. Masereeuw R. (2021). The potential of multi-organ-on-chip models for assessment of drug disposition as alternative to animal testing. Curr. Opin. Toxicol. 27, 8–17. 10.1016/j.cotox.2021.05.001

[B168] Vatine G. D. Barrile R. Workman M. J. Sances S. Barriga B. K. Rahnama M. (2019). Human iPSC-derived blood-brain barrier chips enable disease modeling and personalized medicine applications. Cell Stem Cell 24 (6), 995–1005.e6. 10.1016/j.stem.2019.05.011 31173718

[B169] Vogenberg F. R. Isaacson Barash C. Pursel M. (2010). Personalized medicine: Part 1: Evolution and development into theranostics. P T 35 (10), 560–576. 21037908 PMC2957753

[B170] Vormann M. K. Tool L. M. Ohbuchi M. Gijzen L. van Vught R. Hankemeier T. (2022). Modelling and prevention of acute kidney injury through ischemia and reperfusion in a combined human renal proximal tubule/blood vessel-on-a-chip. Kidney 360 3 (2), 217–231. 10.34067/KID.0003622021 35373131 PMC8967632

[B171] Vormann M. K. Vriend J. Lanz H. L. Gijzen L. van den Heuvel A. Hutter S. (2021). Implementation of a human renal proximal tubule on a chip for nephrotoxicity and drug interaction studies. J. Pharm. Sci. 110 (4), 1601–1614. 10.1016/j.xphs.2021.01.028 33545187

[B172] Wagner K. T. Radisic M. (2021). A new role for extracellular vesicles in cardiac tissue engineering and regenerative medicine. Adv. NanoBiomed Res. 1 (11), 2100047. 10.1002/anbr.202100047 34927167 PMC8680295

[B173] Wang E. Y. Kuzmanov U. Smith J. B. Dou W. Rafatian N. Lai B. F. L. (2021). An organ-on-a-chip model for pre-clinical drug evaluation in progressive non-genetic cardiomyopathy. J. Mol. Cell. Cardiol. 160, 97–110. 10.1016/j.yjmcc.2021.06.012 34216608

[B174] Wang J. Wang C. Xu N. Liu Z.-F. Pang D.-W. Zhang Z.-L. (2019). A virus-induced kidney disease model based on organ-on-a-chip: Pathogenesis exploration of virus-related renal dysfunctions. Biomaterials 219, 119367. 10.1016/j.biomaterials.2019.119367 31344514

[B175] Wiedenmann S. Breunig M. Merkle J. von Toerne C. Georgiev T. Moussus M. (2021). Single-cell-resolved differentiation of human induced pluripotent stem cells into pancreatic duct-like organoids on a microwell chip. Nat. Biomed. Eng. 5 (8), 897–913. 10.1038/s41551-021-00757-2 34239116 PMC7611572

[B176] Wikswo J. P. Curtis E. L. Eagleton Z. E. Evans B. C. Kole A. Hofmeister L. H. (2013). Scaling and systems biology for integrating multiple organs-on-a-chip. Lab a Chip 13 (18), 3496–3511. 10.1039/c3lc50243k PMC3818688 23828456

[B177] Wu J. Dong M. Rigatto C. Liu Y. Lin F. (2018). Lab-on-chip technology for chronic disease diagnosis. npj Digit. Med. 1 (1), 7. 10.1038/s41746-017-0014-0 31304292 PMC6550168

[B178] Yoon J.-K. Kim J. Shah Z. Awasthi A. Mahajan A. Kim Y. (2021). Advanced human BBB-on-a-Chip: A new platform for alzheimer's disease studies. Adv. Healthc. Mater. 10 (15), 2002285. 10.1002/adhm.202002285 PMC8349886 34075728

[B179] Yun S. Choi D. Choi D.-J. Jin S. Yun W.-S. Huh J.-B. (2021). Bone fracture-treatment method: Fixing 3D-printed polycaprolactone scaffolds with hydrogel type bone-derived extracellular matrix and β-tricalcium phosphate as an osteogenic promoter. Int. J. Mol. Sci. 22, 9084. 10.3390/ijms22169084 34445788 PMC8396563

[B180] Zhang C. J. Meyer S. R. O’Meara M. J. Huang S. Capeling M. M. Ferrer-Torres D. (2023). A human liver organoid screening platform for DILI risk prediction. J. Hepatology S0168-8278, 00072–00077. 10.1016/j.jhep.2023.01.019 PMC11268729 36738840

[B181] Zhang J. Chen Z. Zhang Y. Wang X. Ouyang J. Zhu J. (2021b). Construction of a high fidelity epidermis-on-a-chip for scalable *in vitro* irritation evaluation. Lab a Chip 21 (19), 3804–3818. 10.1039/d1lc00099c 34581381

[B182] Zhang J. Wang P. Luo R. Wang Y. Li Z. Guo Y. (2021a). Biomimetic human disease model of SARS-CoV-2-induced lung injury and immune responses on organ chip system. Adv. Sci. 8 (3), 2002928. 10.1002/advs.202002928 PMC7646023 33173719

[B183] Zhang Y. S. Aleman J. Arneri A. Bersini S. Piraino F. Shin S. R. (2015). From cardiac tissue engineering to heart-on-a-chip: Beating challenges. Biomed. Mater. 10 (3), 034006. 10.1088/1748-6041/10/3/034006 26065674 PMC4489846

[B184] Zhang Y. S. Arneri A. Bersini S. Shin S.-R. Zhu K. Goli-Malekabadi Z. (2016). Bioprinting 3D microfibrous scaffolds for engineering endothelialized myocardium and heart-on-a-chip. Biomaterials 110, 45–59. 10.1016/j.biomaterials.2016.09.003 27710832 PMC5198581

[B185] Zhao Y. Rafatian N. Wang E. Y. Wu Q. Lai B. F. L. Lu R. X. (2020). Towards chamber specific heart-on-a-chip for drug testing applications. Adv. Drug Deliv. Rev. 165-166, 60–76. 10.1016/j.addr.2019.12.002 31917972 PMC7338250

[B186] Zheng F. Xiao Y. Liu H. Fan Y. Dao M. (2021). Patient-specific organoid and organ-on-a-chip: 3D cell-culture meets 3D printing and numerical simulation. Adv. Biol. 5 (6), e2000024. 10.1002/adbi.202000024 PMC8243895 33856745

[B187] Zhou H. Hui X. Li D. Hu D. Chen X. He X. (2020a). Metal–organic framework-surface-enhanced infrared absorption platform enables simultaneous on-chip sensing of greenhouse gases. Adv. Sci. 7 (20), 2001173. 10.1002/advs.202001173 PMC7578855 33101855

[B188] Zhou M. Zhang X. Wen X. Wu T. Wang W. Yang M. (2016). Development of a functional glomerulus at the organ level on a chip to mimic hypertensive nephropathy. Sci. Rep. 6 (1), 31771. 10.1038/srep31771 27558173 PMC4997336

[B189] Zhou H. Qu M. Tebon P. Jiang X. Wang C. Xue Y. (2020b). Screening cancer immunotherapy: When engineering approaches meet artificial intelligence. Adv. Sci. 7 (19), 2001447. 10.1002/advs.202001447 PMC7539186 33042756

